# Supplementary material for: Inherited deficiency of DIAPH1 identifies a DNA double strand break repair pathway regulated by γ-actin
Source: Nat Commun. 2025 May 14;16:4491. doi: 10.1038/s41467-025-59553-0 (PMC12078678; doi:10.1038/s41467-025-59553-0)
Supplement: Supplementary file 1 — Supplementary Information [file 41467_2025_59553_MOESM1_ESM.pdf]

a

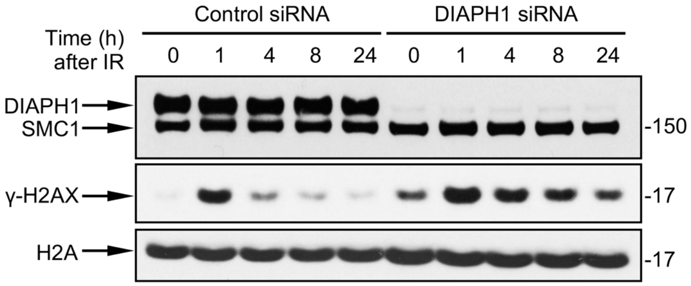

Control siRNA
DIAPH1 siRNA
Time (h) after IR
0
1
4
8
24
0
1
4
8
24
DIAPH1
SMC1
-150
γ-H2AX
-17
H2A
-17

b

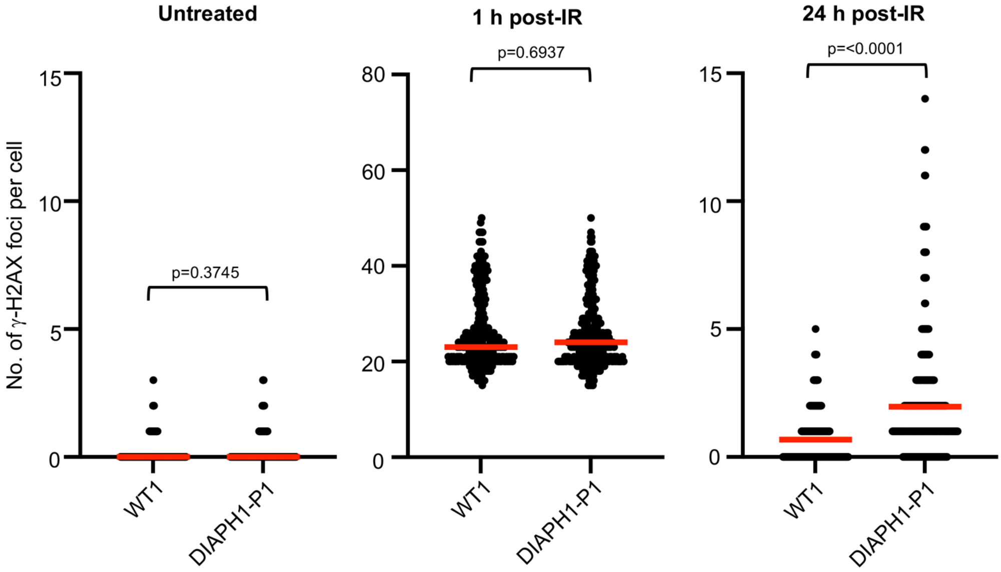

Untreated
1 h post-IR
24 h post-IR
No. of γ-H2AX foci per cell
p=0.3745
p=0.6937
p=<0.0001
0
5
10
15
0
20
40
60
80
WT1
DIAPH1-P1

Supplementary Fig. 1. Cells lacking DIAPH1 display an inability to repair DSBs induced by IR. **a** HeLa cells were transfected with either control siRNA or an siRNA targeting DIAPH1, and then mock treated or irradiated with 3 Gy of IR. Cells were harvested for Western blotting at the time points post-irradiation indicated. Cell extracts were separated by SDS-PAGE and Western blotting was carried out with the antibodies indicated. Representative of n=2 independent experiments. **b** Quantification of γ-H2AX foci in WT and patient P1 fibroblasts before and after exposure to 1 Gy IR. γ-H2AX foci were visualised by immunofluorescence microscopy and quantified in untreated cells and cells 1 h and 24 h following IR exposure. The mean of n=3 independent experiments is shown with the SEM (red line). γ-H2AX foci were counted in 100 cells per time point, per experiment. Statistical significance was calculated using: (b) a Mann-Whitney rank sum test. Source data are provided as a Source Data file.

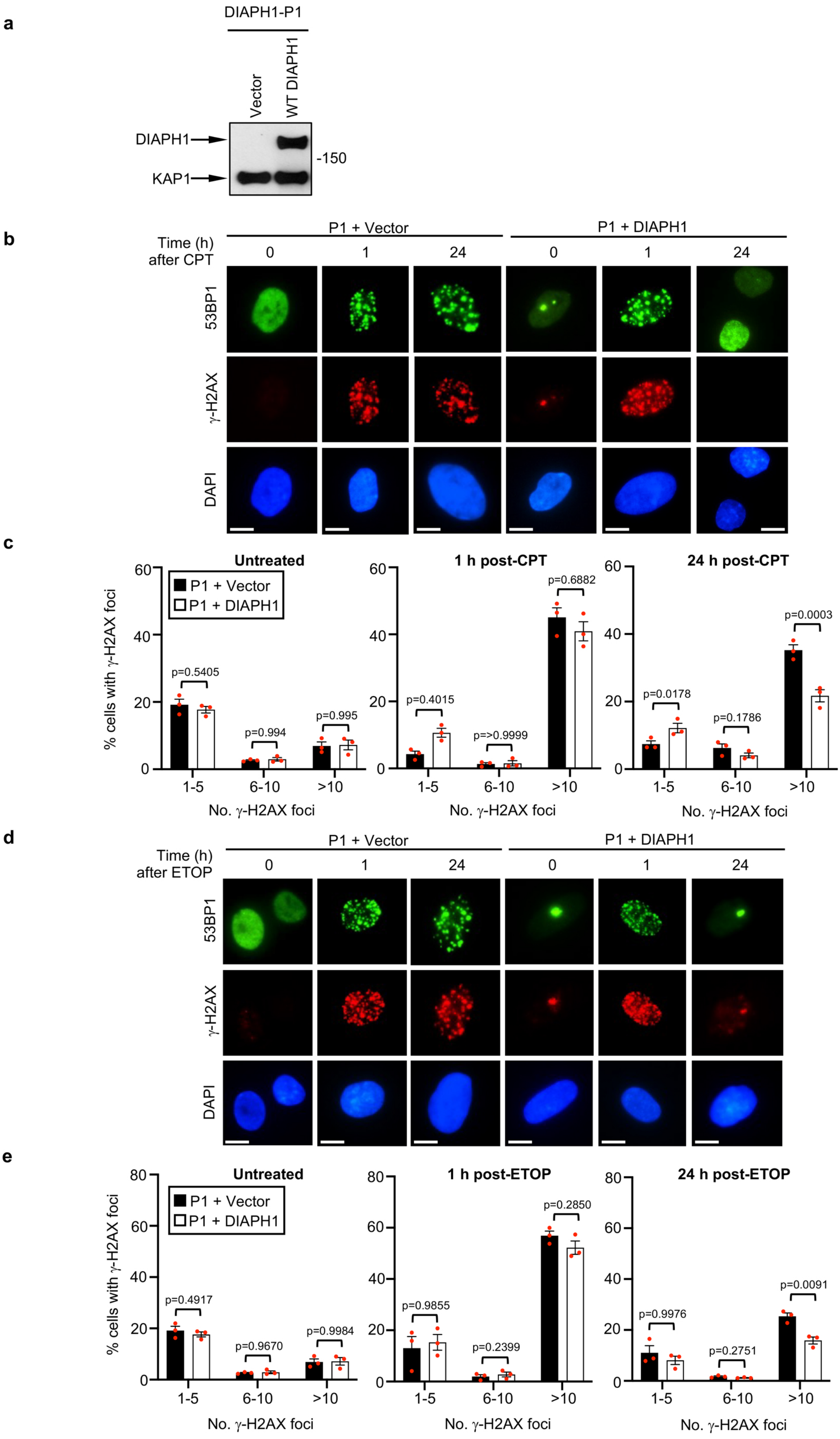

a
DIAPH1-P1
Vector
WT DIAPH1
DIAPH1
KAP1
-150
b
Time (h) after CPT
P1 + Vector
P1 + DIAPH1
0
1
24
53BP1
γ-H2AX
DAPI
c
Untreated
1 h post-CPT
24 h post-CPT
P1 + Vector
P1 + DIAPH1
% cells with γ-H2AX foci
No. γ-H2AX foci
1-5
6-10
>10
p=0.5405
p=0.994
p=0.995
p=0.4015
p=>0.9999
p=0.6882
p=0.0178
p=0.1786
p=0.0003
d
Time (h) after ETOP
P1 + Vector
P1 + DIAPH1
0
1
24
53BP1
γ-H2AX
DAPI
e
Untreated
1 h post-ETOP
24 h post-ETOP
P1 + Vector
P1 + DIAPH1
% cells with γ-H2AX foci
No. γ-H2AX foci
1-5
6-10
>10
p=0.4917
p=0.9670
p=0.9984
p=0.9855
p=0.2399
p=0.2850
p=0.9976
p=0.2751
p=0.0091

Supplementary Fig. 2. Cells lacking DIAPH1 display an inability to repair DSBs induced by CPT and ETOP.

a A Western blot of DIAPH1 expression in cell extracts from DIAPH1-P1 fibroblasts complemented with either an empty vector or a vector expressing WT *DIAPH1*. KAP1 is used as a protein loading control. n=1 independent experiments. **b-c** Quantification of γ-H2AX foci in complemented DIAPH1-P1 fibroblasts before and after exposure to 100 nM CPT for 1 h. γ-H2AX/53BP1 foci were visualised by immunofluorescence microscopy and quantified in untreated cells and cells 1 h and 24 h following CPT treatment. The mean of n=3 independent experiments is shown with the SEM. A minimum of 500 cells were counted per time point, per experiment. Representative images of the cells at different time points before and post-treatment are shown (b). The scale bars represent 10 μm. **d-e** Quantification of γ-H2AX foci in complemented DIAPH1-P1 fibroblasts before and after exposure to 10 μM ETOP for 30 min. γ-H2AX/53BP1 foci were visualised by immunofluorescence microscopy and quantified in untreated cells and cells 1 h and 24 h following ETOP treatment. The mean of n=3 independent experiments is shown with the SEM. A minimum of 500 cells were counted per time point, per experiment. Representative images of the cells at different time points before and post-treatment are shown (d). The scale bars represent 10 μm. Note: The quantification of γ-H2AX foci for CPT (c) and ETOP (e) treated cells were carried out as part of the same experiment using the same untreated controls. Therefore, the quantification of γ-H2AX foci in untreated cells in (c) has been duplicated in (e) so that comparisons with the quantification of γ-H2AX foci in ETOP-treated cells can be made. Statistical significance was calculated using: (c & e) an two-way ANOVA with multiple comparisons. Source data are provided as a Source Data file.

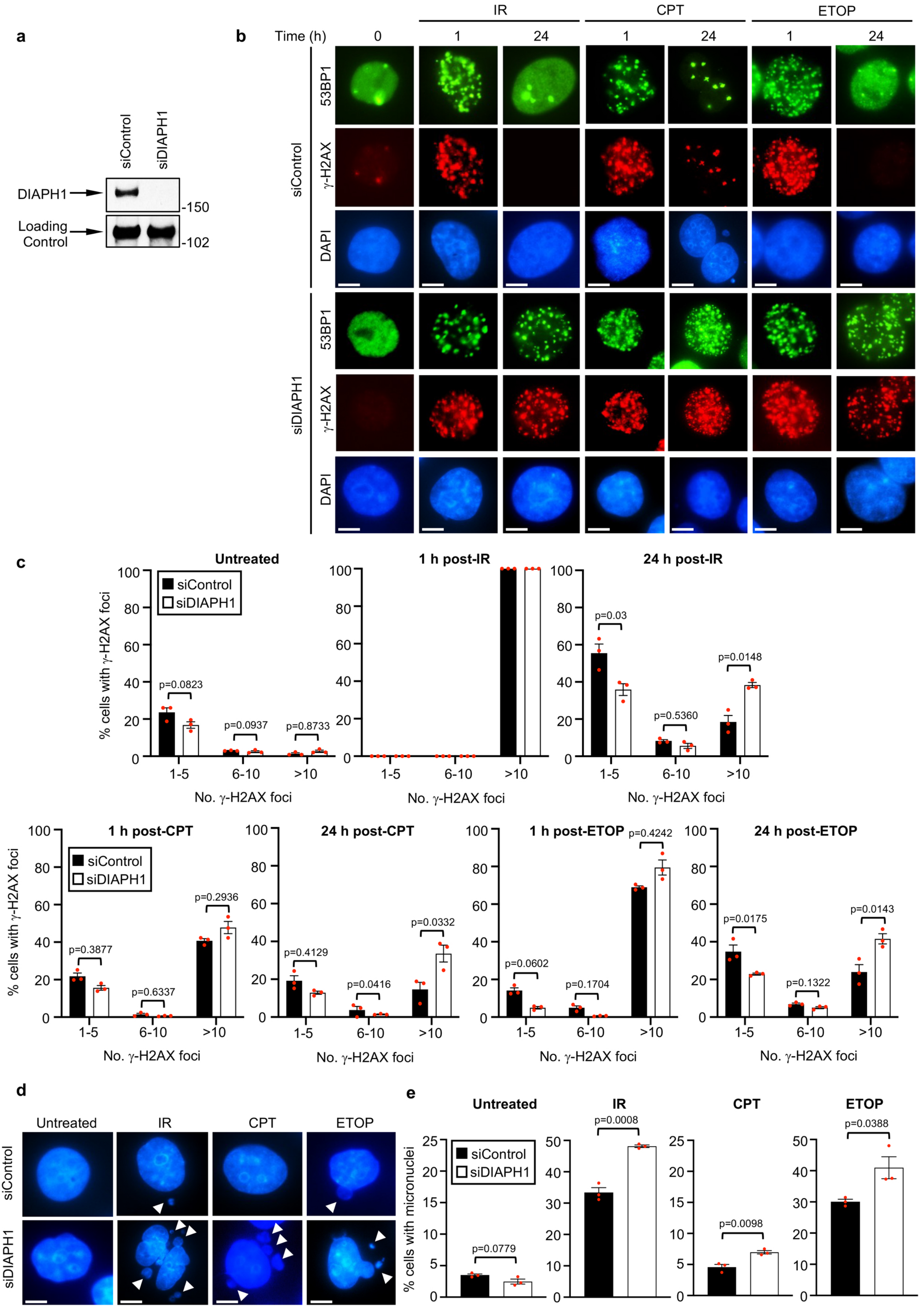
a
siControl
siDIAPH1
DIAPH1
-150
Loading Control
-102
b
Time (h)
IR
CPT
ETOP
0
1
24
1
24
1
24
siControl
siDIAPH1
53BP1
γ-H2AX
DAPI
c
Untreated
1 h post-IR
24 h post-IR
1 h post-CPT
24 h post-CPT
1 h post-ETOP
24 h post-ETOP
siControl
siDIAPH1
% cells with γ-H2AX foci
No. γ-H2AX foci
1-5
6-10
>10
p=0.0823
p=0.0937
p=0.8733
p=0.03
p=0.5360
p=0.0148
p=0.3877
p=0.6337
p=0.2936
p=0.4129
p=0.0416
p=0.0332
p=0.0602
p=0.1704
p=0.4242
p=0.0175
p=0.1322
p=0.0143
d
Untreated
IR
CPT
ETOP
siControl
siDIAPH1
e
Untreated
IR
CPT
ETOP
% cells with micronuclei
p=0.0779
p=0.0008
p=0.0098
p=0.0388

Supplementary Fig. 3. Cells depleted of DIAPH1 display an inability to repair DSBs induced by IR, CPT and ETOP.

a A Western blot of DIAPH1 expression in extracts from HeLa cells transfected with control or DIAPH1 targeting siRNA. A cross-reactive band is used as a protein loading control. n=1 independent experiments. **b-c** Quantification of γ-H2AX foci in HeLa cells transfected with control or DIAPH1 targeting siRNA before and after exposure to 3 Gy IR, 100 nM CPT for 1 h or 10 μM ETOP for 30 min. γ-H2AX/53BP1 foci were visualised by immunofluorescence microscopy and quantified in untreated cells and cells 1 h and 24 h following IR, CPT or ETOP treatment. The mean of n=3 independent experiments is shown with the SEM. A minimum of 500 cells were counted per time point, per experiment. Representative images of the cells at different time points before and post-treatment are shown (b). The scale bars represent 10 μm. **d-e** Micronuclei were quantified from HeLa cells described in (a) before and after exposure to IR (24 h), CPT (48 h) and ETOP (48 h). The mean of n=3 independent experiments is shown with the SEM. A minimum of 500 cells were counted per time point, per experiment. Representative images of the cells at different time points before and after treatment are shown (d). The scale bars represent 10 μm. Statistical significance was calculated using: (c) an two-way ANOVA with multiple comparisons and (e) an unpaired Student's t-test (two-sided, equal variance). Source data are provided as a Source Data file.

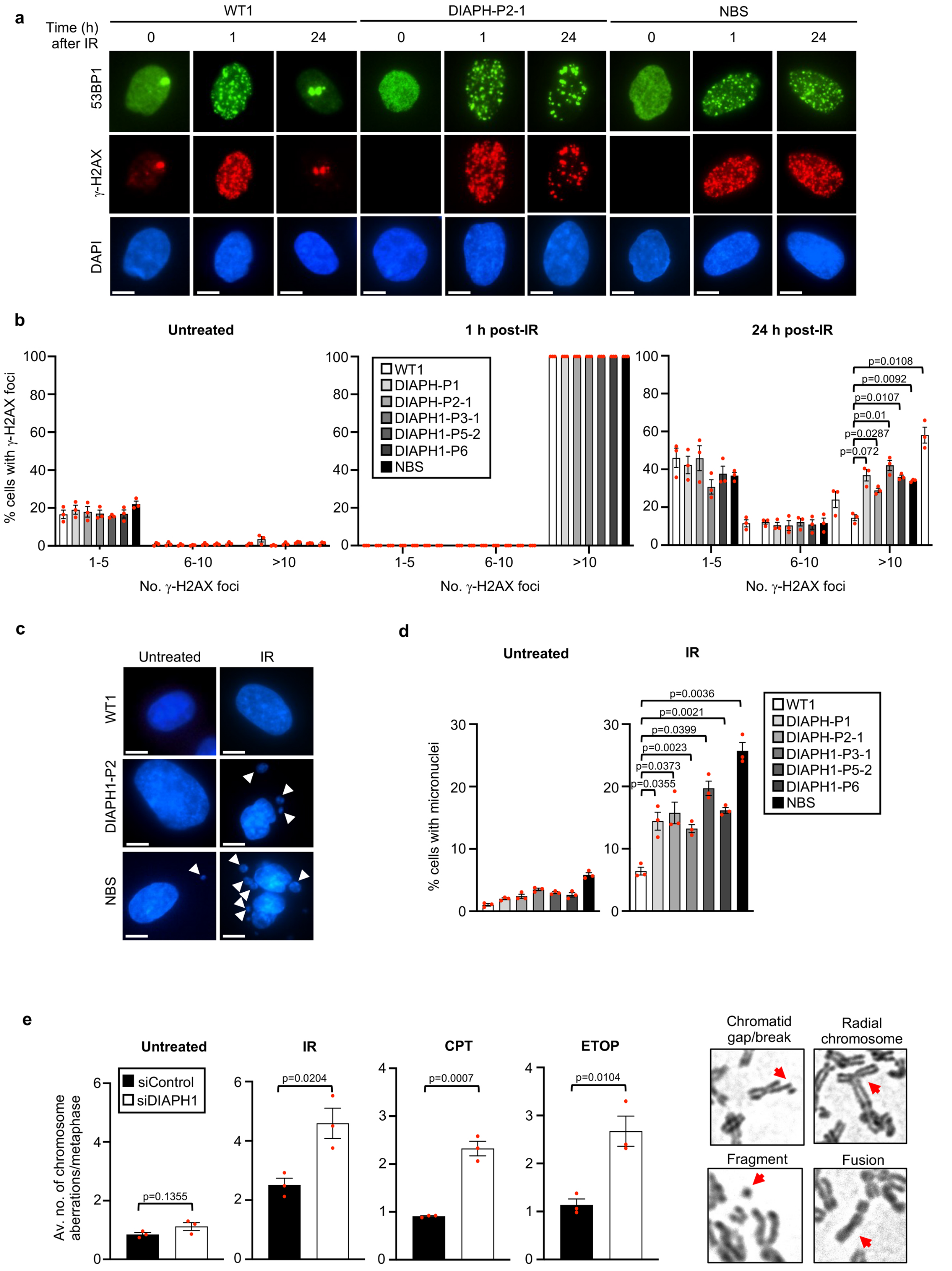

a
Time (h) after IR
WT1
DIAPH-P2-1
NBS
0
1
24
53BP1
γ-H2AX
DAPI
b
Untreated
1 h post-IR
24 h post-IR
% cells with γ-H2AX foci
No. γ-H2AX foci
1-5
6-10
>10
WT1
DIAPH-P1
DIAPH-P2-1
DIAPH1-P3-1
DIAPH1-P5-2
DIAPH1-P6
NBS
p=0.0108
p=0.0092
p=0.0107
p=0.01
p=0.0287
p=0.072
c
Untreated
IR
WT1
DIAPH1-P2
NBS
d
Untreated
IR
% cells with micronuclei
p=0.0036
p=0.0021
p=0.0399
p=0.0023
p=0.0373
p=0.0355
WT1
DIAPH-P1
DIAPH-P2-1
DIAPH1-P3-1
DIAPH1-P5-2
DIAPH1-P6
NBS
e
Untreated
IR
CPT
ETOP
Av. no. of chromosome aberrations/metaphase
siControl
siDIAPH1
p=0.1355
p=0.0204
p=0.0007
p=0.0104
Chromatid gap/break
Radial chromosome
Fragment
Fusion

Supplementary Fig. 4. Analysis of DSBR in five independent DIAL syndrome patient-derived cell lines using immunofluorescence and chromosome breakage analysis.

a-b Quantification of γ-H2AX foci in WT fibroblasts (WT1), fibroblasts derived from a patient with Nijmegen Breakage Syndrome (NBS) and fibroblasts derived from 5 different DIAL syndrome patients (P1, P2-1, P3-1, P5-2 and P6) before and after exposure to 3 Gy IR. γ-H2AX foci were visualised by immunofluorescence microscopy and quantified in untreated cells and cells 1 h and 24 h post-irradiation. The mean of n=3 independent experiments is shown with the SEM. A minimum of 500 cells were counted per time point, per experiment. Representative images of the cells at different time points before and post-irradiation are shown (a). The scale bars represent 10 μm. **c-d** Micronuclei were quantified from cells described in (a-b) before and after exposure to IR. The mean of n=3 independent experiments is shown with the SEM. A minimum of 500 cells were counted per time point, per experiment. Representative images of the cells at different time points before and after treatment are shown (c). The scale bars represent 10 μm. **e** Quantification of chromosome aberrations in HeLa cells transfected with control siRNA or an siRNA targeting DIAPH1 before and 24 h after 2 Gy of IR or chronic exposure to low dose CPT (5 nM) or ETOP (50 nM). Chromosome aberrations includes chromatid/chromosome gaps/breaks, chromatid/chromosome fragments and chromosome radials/exchanges. Representative images of each type of aberration are shown. The mean of n=3 independent experiments is shown with the SEM. A minimum of 50 metaphases were counted per cell line in each experiment. Statistical significance was calculated using: (b) a two-way ANOVA with multiple comparisons, (d) an ordinary one-way ANOVA (p=<0.0001) and (e) an unpaired Student's t-test (two-sided, equal variance). Source data are provided as a Source Data file.

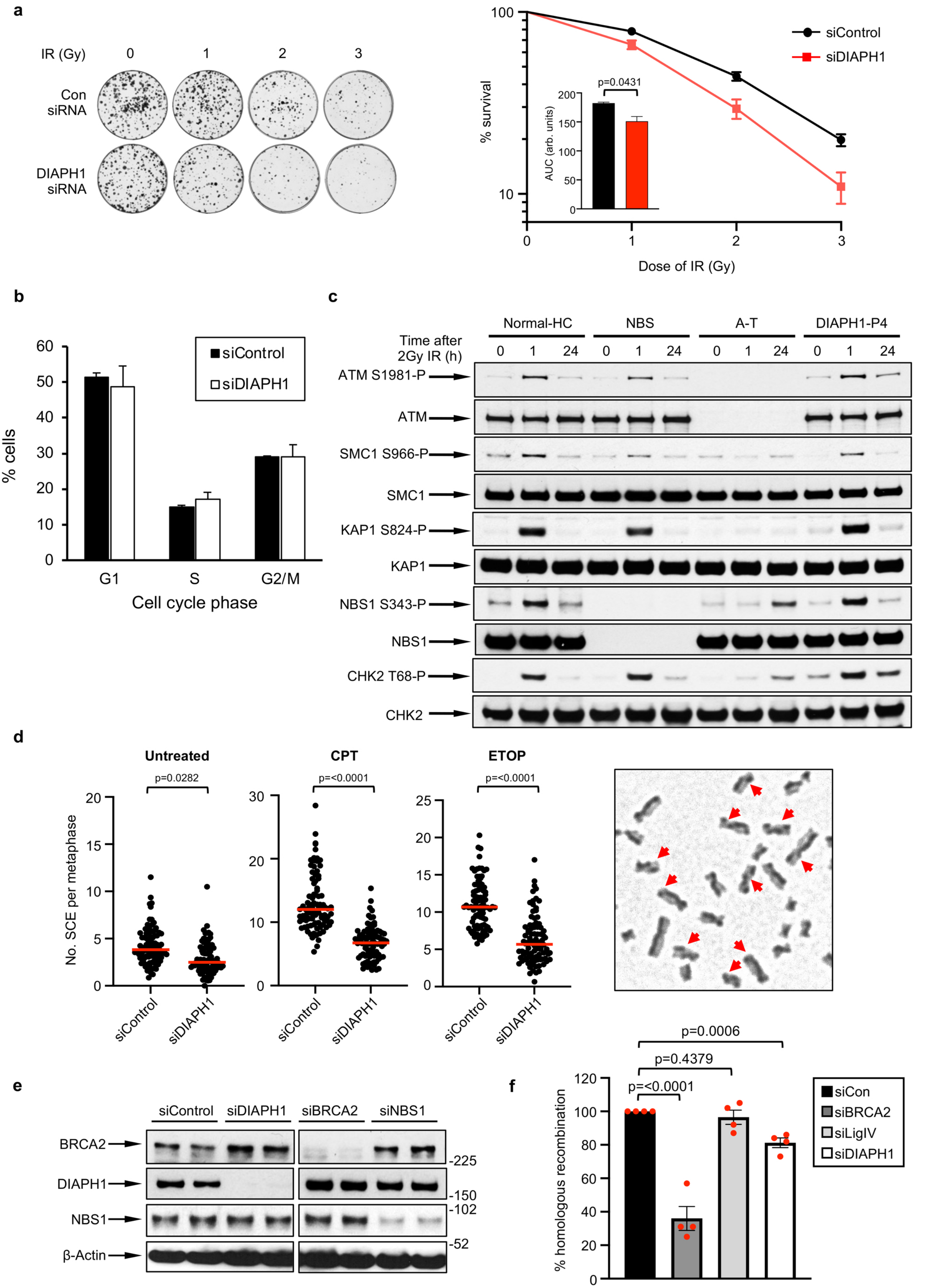

a
IR (Gy)
0
1
2
3
Con siRNA
DIAPH1 siRNA
% survival
Dose of IR (Gy)
siControl
siDIAPH1
AUC (arb. units)
p=0.0431
b
% cells
siControl
siDIAPH1
G1
S
G2/M
Cell cycle phase
c
Normal-HC
NBS
A-T
DIAPH1-P4
Time after 2Gy IR (h)
ATM S1981-P
ATM
SMC1 S966-P
SMC1
KAP1 S824-P
KAP1
NBS1 S343-P
NBS1
CHK2 T68-P
CHK2
d
Untreated
CPT
ETOP
p=0.0282
p=<0.0001
p=<0.0001
No. SCE per metaphase
siControl
siDIAPH1
e
siControl
siDIAPH1
siBRCA2
siNBS1
BRCA2
DIAPH1
NBS1
β-Actin
-225
-150
-102
-52
f
% homologous recombination
p=<0.0001
p=0.4379
p=0.0006
siCon
siBRCA2
siLigIV
siDIAPH1

Supplementary Fig. 5. DIAL syndrome cells do not exhibit any defects in activation of the ATM-dependent DDR following exposure to IR.

a U-2 OS cells transfected with either control or DIAPH1 siRNA were plated at low density, exposed to the doses of IR indicated and were allowed to form colonies for 14 days. Colonies were fixed, stained and counted. Representative images of cell colonies are shown (left). Colonies were normalized to the number of cells plated and expressed as a mean % cell survival relative to the untreated control. Quantification of colonies from n=3 independent experiments. **b** Quantification of cell cycle distribution of U-2 OS cells in part (a) as determined by propidium iodide staining coupled with flow cytometry. n=3 independent experiments. **c** Western blot on cell extracts derived from LCLs from one WT individual (Normal-HC), an individual with NBS, an individual with A-T and DIAPH1 patient P5 before and after exposure to 2 Gy of IR. Timepoints were taken 1 h and 24 h post-irradiation. Antibodies used for Western blotting are indicated. Phospho-specific antibodies are denoted with the position of the phosphorylated residue followed by a 'P'. Antibodies to non-phosphorylated proteins were used to control for protein loading. All Western blots using phospho-specific antibodies were generated from cutting the same nitrocellulose filter into strips and blotting with the antibodies indicated. All Western blots using antibodies to non-phosphorylated proteins were generated by running the same samples in parallel on a separate gel, cutting the nitrocellulose filter into strips and blotting with the antibodies indicated. Representative of n=2 independent experiments. **d** Quantification SCEs in HeLa cells transfected with control and DIAPH1 siRNA before and 24 h after chronic exposure to low dose CPT (0.5 nM) and ETOP (10 nM). The mean of n=3 independent experiments is shown (red line). Approximately 30 metaphases per cell line were scored for SCEs per experiment. A representative image of the SCEs quantified is shown. **e** Efficiency of siRNA-mediated gene knockdown in HeLa cells for Fig. 3c. n=1 independent experiments. **f** Quantification of the relative levels of HR-dependent of an I-SceI-induced DSB using the DR-GFP assay in cells when either BRCA2, DNA ligase IV or DIAPH1 are depleted using siRNA. The mean of n=4 independent experiments is shown. The relative levels of HR were normalised in all samples to that observed in the control siRNA (siCon) transfected cells. Statistical significance was calculated using: (a) a paired Student's t-test, (d) unpaired Student's t-test (two-sided, equal variance) and (f) an ordinary one-way ANOVA. Source data are provided as a Source Data file.

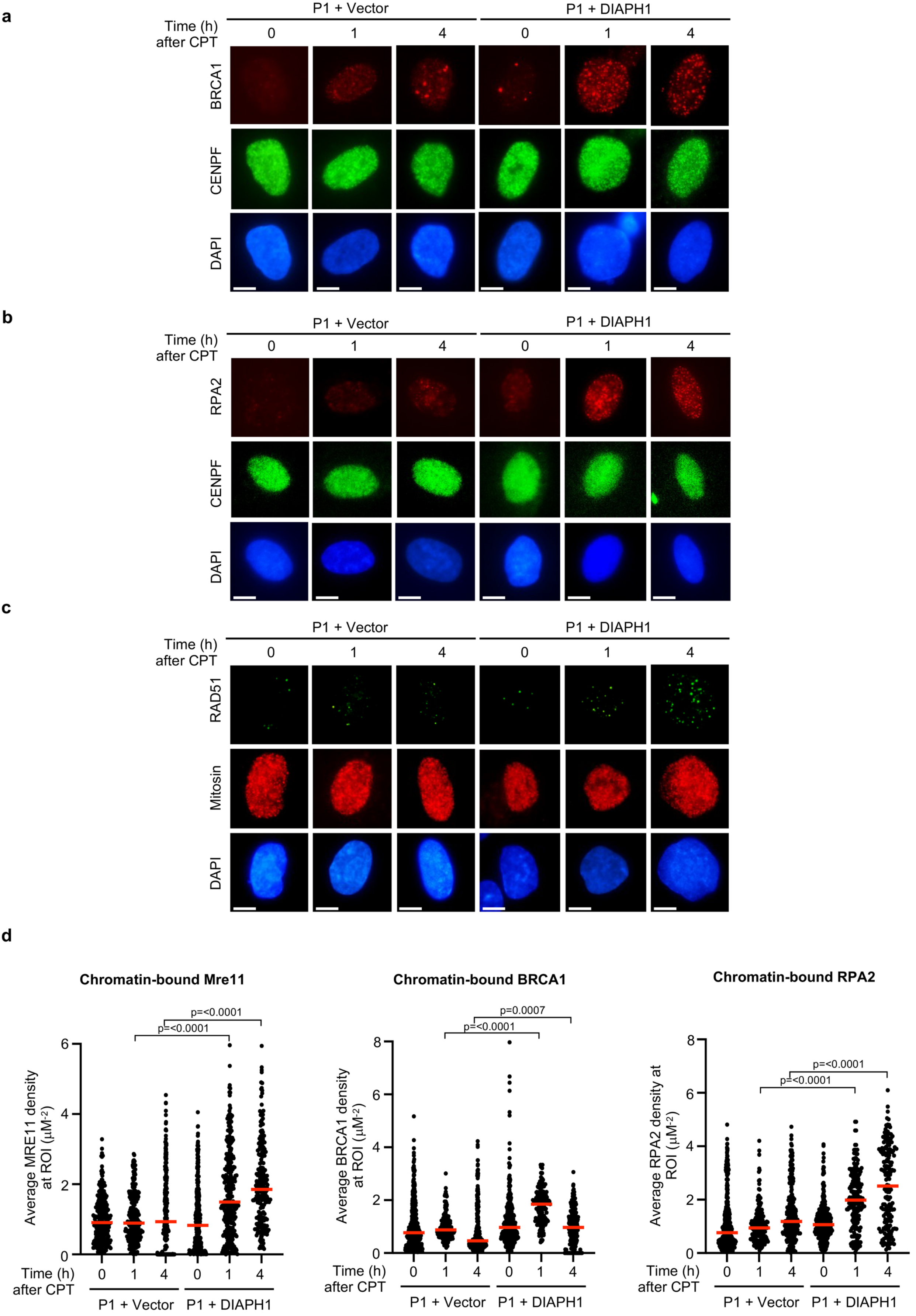
a
P1 + Vector
P1 + DIAPH1
Time (h) after CPT
0
1
4
0
1
4
BRCA1
CENPF
DAPI
b
P1 + Vector
P1 + DIAPH1
Time (h) after CPT
0
1
4
0
1
4
RPA2
CENPF
DAPI
c
P1 + Vector
P1 + DIAPH1
Time (h) after CPT
0
1
4
0
1
4
RAD51
Mitosin
DAPI
d
Chromatin-bound Mre11
Average MRE11 density at ROI (μM-2)
p=<0.0001
p=<0.0001
Time (h) after CPT
0
1
4
0
1
4
P1 + Vector
P1 + DIAPH1
Chromatin-bound BRCA1
Average BRCA1 density at ROI (μM-2)
p=0.0007
p=<0.0001
Time (h) after CPT
0
1
4
0
1
4
P1 + Vector
P1 + DIAPH1
Chromatin-bound RPA2
Average RPA2 density at ROI (μM-2)
p=<0.0001
p=<0.0001
Time (h) after CPT
0
1
4
0
1
4
P1 + Vector
P1 + DIAPH1

Supplementary Fig. 6. Representative images for BRCA1, RPA2 and RAD51 foci in S/G2-phase complemented DIAL syndrome cells following exposure to CPT.

a-c Representative images of the complemented DIAL syndrome cells used for the quantification of CPT-induced (a) BRCA1, (b) RPA2 and (c) RAD51 foci in Fig. 3d-f. The scale bars represent 10 μm. **d** Quantification of the amount of CPT-induced chromatin retention of MRE11 (left), BRCA1 (middle) and RPA2 (right) within the ROIs indicated in Fig. 4a-b. A minimum of 100 ROIs were quantified over three independent experiments. The median of n=3 independent experiments is shown (red line). Statistical significance was calculated using: (d) a Kruskal-Wallis test ([chromatin-bound MRE11] p=<0.0001, [chromatin-bound BRCA1] p=0.0007, [chromatin-bound RPA2] p=<0.0001). Source data are provided as a Source Data file.

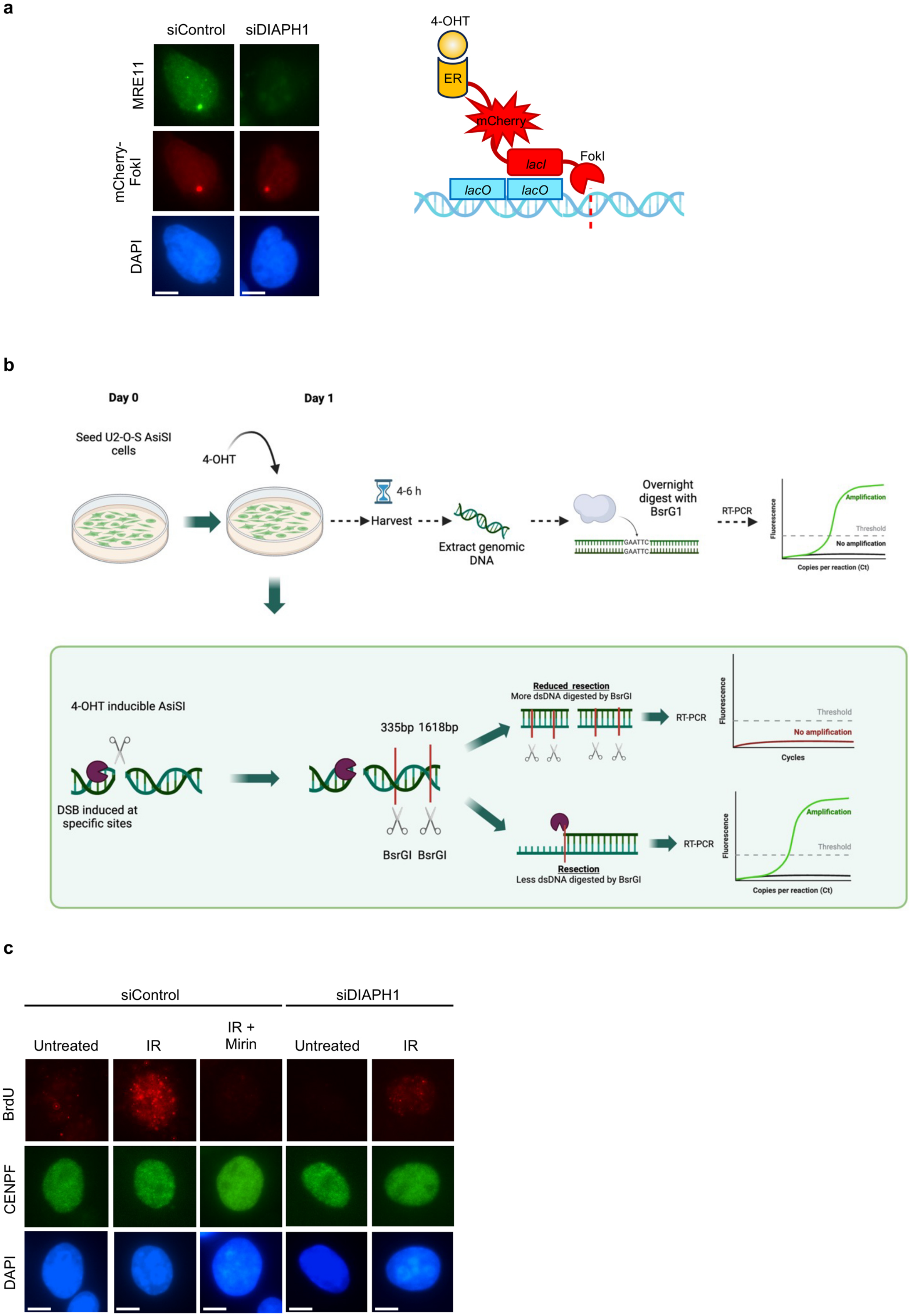
a
siControl
siDIAPH1
MRE11
mCherry-FokI
DAPI
4-OHT
ER
mCherry
lacI
FokI
lacO
lacO
b
Day 0
Day 1
Seed U2-O-S AsiSI cells
4-OHT
4-6 h
Harvest
Extract genomic DNA
Overnight digest with BsrG1
GAATTC
GAATTC
RT-PCR
Fluorescence
Amplification
Threshold
No amplification
Copies per reaction (Ct)
4-OHT inducible AsiSI
DSB induced at specific sites
335bp 1618bp
BsrGI BsrGI
Reduced resection
More dsDNA digested by BsrGI
RT-PCR
Fluorescence
Threshold
No amplification
Cycles
Resection
Less dsDNA digested by BsrGI
RT-PCR
Fluorescence
Amplification
Threshold
Copies per reaction (Ct)
c
siControl
siDIAPH1
Untreated
IR
IR + Mirin
Untreated
IR
BrdU
CENPF
DAPI

Supplementary Fig. 7. Representative images for MRE11 localisation to FokI-induced DSBs and CPT-induced DNA end-resection in cells with/without DIAPH1.

a (Left) Representative images used for the quantification of MRE11 recruitment to FokI-induced DNA DSBs in U-2 OS cells transfected with control or DIAPH1 siRNA (Fig. 4c). The scale bars represent 10 μm. (Right) A diagrammatic representation of how the ER-mCherry-LacI-FokI-DD system induces a DSB. ER: estrogen-receptor; 4-OHT: 4-hydroxy-tamoxifen; *lacO*: lactose operon; *lacI*: lac repressor. **b** Diagrammatic representation of how resection of an *Asi*S1-induced DSB on chromosome 1 is quantified. Created in BioRender. Woodward, B. (2025) https://BioRender.com/jij9l1g. **c** Representative images used for the quantification of DSB end-resection as judged by native BrdU foci in HeLa cells transfected with control or DIAPH1 siRNA following exposure to 100 nM CPT for 1 h and permeabilised/fixed 4 h post-treatment (Fig. 4e). The scale bars represent 10 μm.

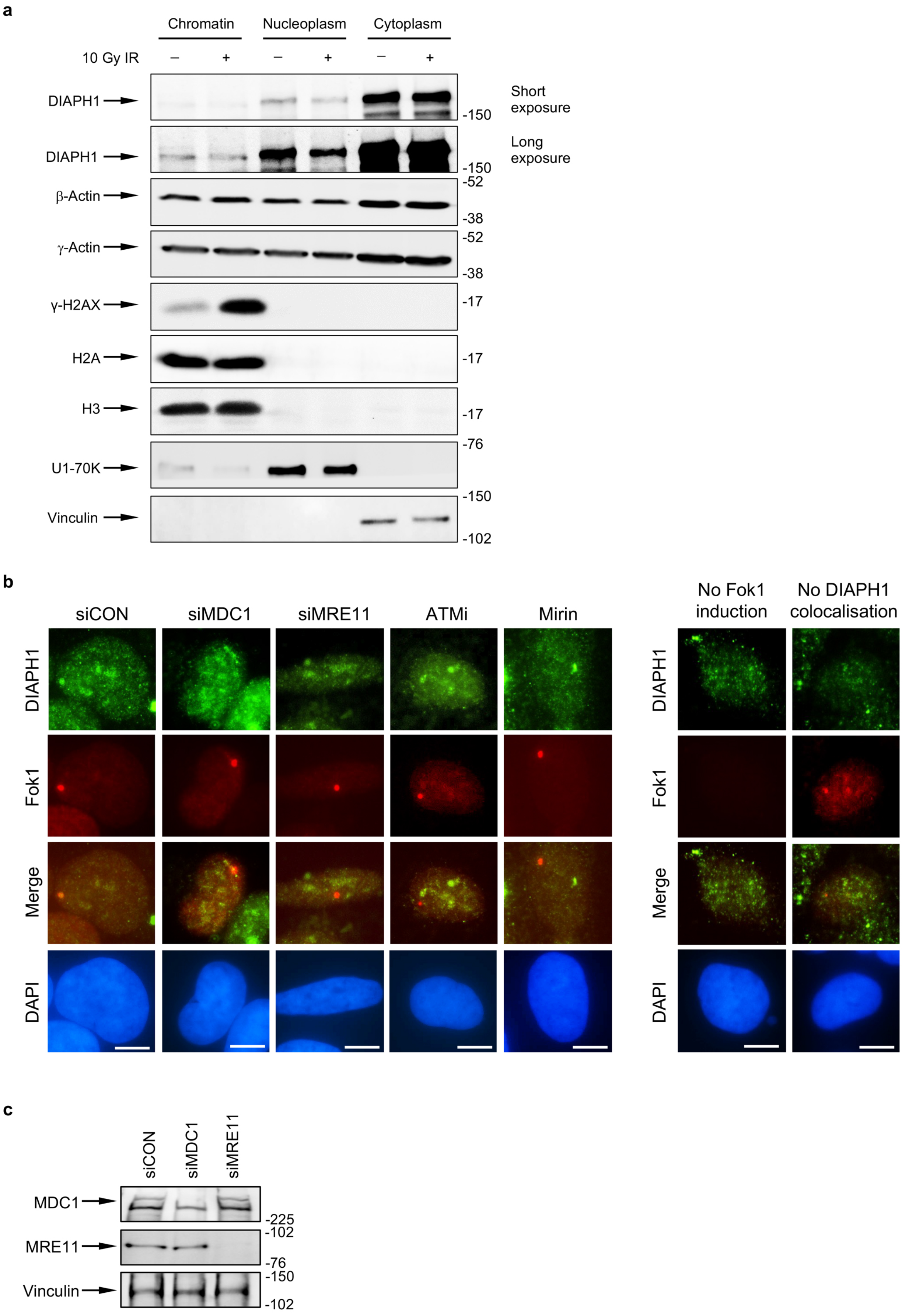

a
Chromatin
Nucleoplasm
Cytoplasm
10 Gy IR
–
+
DIAPH1
Short exposure
Long exposure
-150
β-Actin
γ-Actin
-52
-38
γ-H2AX
H2A
H3
-17
U1-70K
-76
Vinculin
-150
-102
b
siCON
siMDC1
siMRE11
ATMi
Mirin
No Fok1 induction
No DIAPH1 colocalisation
DIAPH1
Fok1
Merge
DAPI
c
siCON
siMDC1
siMRE11
MDC1
-225
MRE11
-102
-76
Vinculin
-150
-102

Supplementary Fig. 8. DIAPH1 is present in both the nucleus and cytoplasm.

a Cell fractionation assessing the localisation of DIAPH1, γ-actin and β-actin before and 1 h after exposure to 10 Gy of IR. Vinculin, U1-70K and histone H3 are used as markers of the cytoplasmic, nuclear and chromatin fractions respectively. Representative of n=2 independent experiments. **b** (Left) Representative images of the recruitment of DIAPH1 to FokI-induced DNA DSBs in cells depleted of MDC1 or MRE11 or treated with an ATM inhibitor or mirin (Fig. 5d). (Right) Representative images of DIAPH1 localisation in the absence of FokI-induction and cells where DIAPH1 did not localise to FokI-induced DSBs. **c** Western blots showing the efficiency of siRNA-mediated gene knockdown. n=1 independent experiments. The scale bars represent 10 μm.

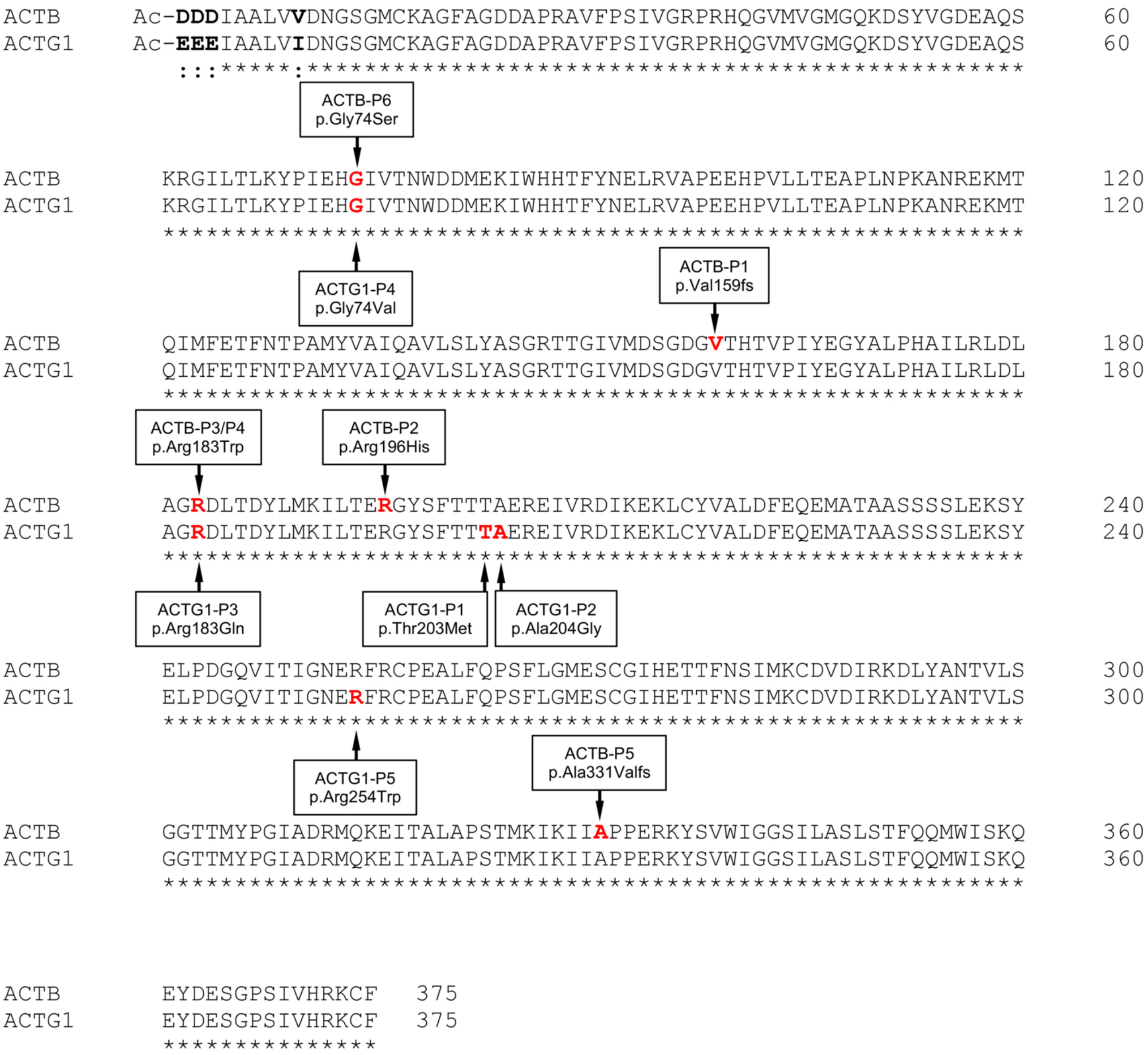
ACTB Ac-DDDIAALVVDNGSGMCKAGFAGDDAPRAVFPSIVGRPRHQGVMVGMGQKDSYVGDEAQS 60
ACTG1 Ac-EEEIAALVIDNGSGMCKAGFAGDDAPRAVFPSIVGRPRHQGVMVGMGQKDSYVGDEAQS 60
:::*****:***
ACTB-P6
p.Gly74Ser
ACTB KRGILTLKYPIEHGIVTNWDDMEKIWHHTFYNELRVAPEEHPVLLTEAPLNPKANREKMT 120
ACTG1 KRGILTLKYPIEHGIVTNWDDMEKIWHHTFYNELRVAPEEHPVLLTEAPLNPKANREKMT 120
****
ACTG1-P4
p.Gly74Val
ACTB-P1
p.Val159fs
ACTB QIMFETFNTPAMYVAIQAVLSLYASGRTTGIVMDSGDGVTHTVPIYEGYALPHAILRLDL 180
ACTG1 QIMFETFNTPAMYVAIQAVLSLYASGRTTGIVMDSGDGVTHTVPIYEGYALPHAILRLDL 180
****
ACTB-P3/P4
p.Arg183Trp
ACTB-P2
p.Arg196His
ACTB AGRDLTDYLMKILTERGYSFTTTAEREIVRDIKEKLCYVALDFEQEMATAASSSSLEKSY 240
ACTG1 AGRDLTDYLMKILTERGYSFTTTAEREIVRDIKEKLCYVALDFEQEMATAASSSSLEKSY 240
****
ACTG1-P3
p.Arg183Gln
ACTG1-P1
p.Thr203Met
ACTG1-P2
p.Ala204Gly
ACTB ELPDGQVITIGNERFRCPEALFQPSFLGMESCGIHETTFNSIMKCDVDIRKDLYANTVLS 300
ACTG1 ELPDGQVITIGNERFRCPEALFQPSFLGMESCGIHETTFNSIMKCDVDIRKDLYANTVLS 300
****
ACTG1-P5
p.Arg254Trp
ACTB-P5
p.Ala331Valfs
ACTB GGTTMYPGIADRMQKEITALAPSTMKIKIIAPPERKYSVWIGGSILASLSTFQQMWISKQ 360
ACTG1 GGTTMYPGIADRMQKEITALAPSTMKIKIIAPPERKYSVWIGGSILASLSTFQQMWISKQ 360
****
ACTB EYDESGPSIVHRKCF 375
ACTG1 EYDESGPSIVHRKCF 375
****

Supplementary Fig. 9. Alignment of the β- and γ-actin protein sequences.

Protein sequence alignment of human β- and γ-actin with the position of all the BWCFF syndrome patient-associated mutations analysed highlighted in red. The four N-terminal amino acids that differ between β- and γ-actin are highlighted in bold. ‘fs’ indicates frameshift.

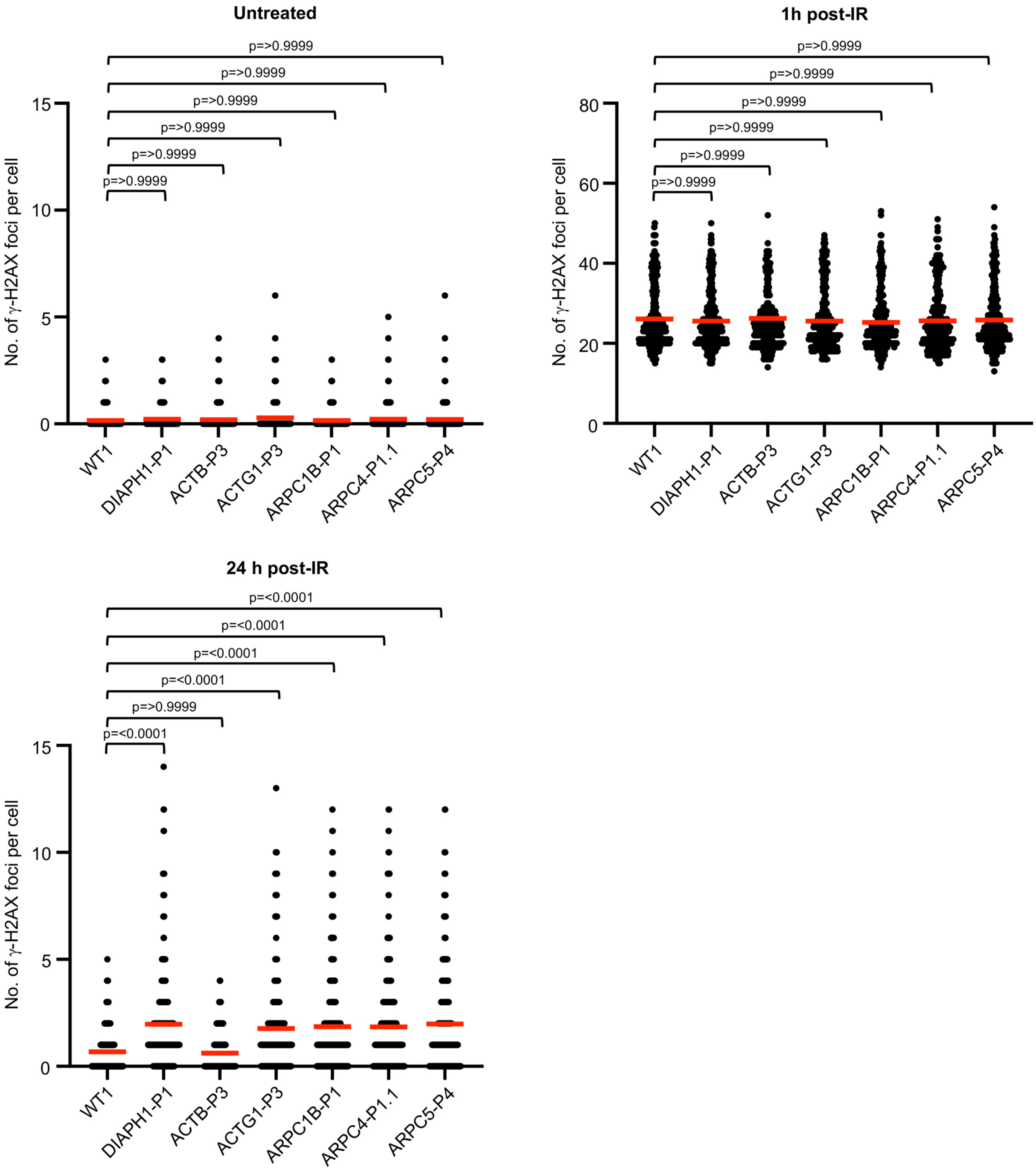
Untreated
p=>0.9999
p=>0.9999
p=>0.9999
p=>0.9999
p=>0.9999
p=>0.9999
No. of γ-H2AX foci per cell
15
10
5
0
WT1
DIAPH1-P1
ACTB-P3
ACTG1-P3
ARPC1B-P1
ARPC4-P1.1
ARPC5-P4
1h post-IR
p=>0.9999
p=>0.9999
p=>0.9999
p=>0.9999
p=>0.9999
p=>0.9999
No. of γ-H2AX foci per cell
80
60
40
20
0
WT1
DIAPH1-P1
ACTB-P3
ACTG1-P3
ARPC1B-P1
ARPC4-P1.1
ARPC5-P4
24 h post-IR
p=<0.0001
p=<0.0001
p=<0.0001
p=<0.0001
p=>0.9999
p=<0.0001
No. of γ-H2AX foci per cell
15
10
5
0
WT1
DIAPH1-P1
ACTB-P3
ACTG1-P3
ARPC1B-P1
ARPC4-P1.1
ARPC5-P4

Supplementary Fig. 10. Cells with mutations in *ACTG1* or the ARP2/3 complex display an inability to repair DSBs induced by IR.

Quantification of γ-H2AX foci in WT fibroblasts and *DIAPH1*, *ACTB*, *ACTG1*, *ARCP1B*, *ARPC4* and *ARPC5* mutant patient fibroblasts before and after exposure to 1 Gy IR. γ-H2AX foci were visualised by immunofluorescence microscopy and quantified in untreated cells and cells 1 h and 24 h following IR exposure. The mean of n=3 independent experiments is shown with the SEM (red line). γ-H2AX foci were counted in 100 cells per time point, per experiment. Statistical significance was calculated using a Kruskal-Wallis test ([untreated] p=0.7591, [1 h post-IR] p=0.0757, [24 h post-IR] p=<0.0001). Note: The quantification of γ-H2AX foci for the WT and DIAPH1-P1 fibroblasts has been duplicated from Supplementary Fig. 1b for comparison purposes. γ-H2AX foci quantification in fibroblasts for all genotypes were performed at the same time as part of the same experimental set. The γ-H2AX foci quantification in WT and DIAPH1-P1 fibroblasts was separated into Supplementary Fig. 1b to fit with the narrative of the manuscript. Source data are provided as a Source Data file.

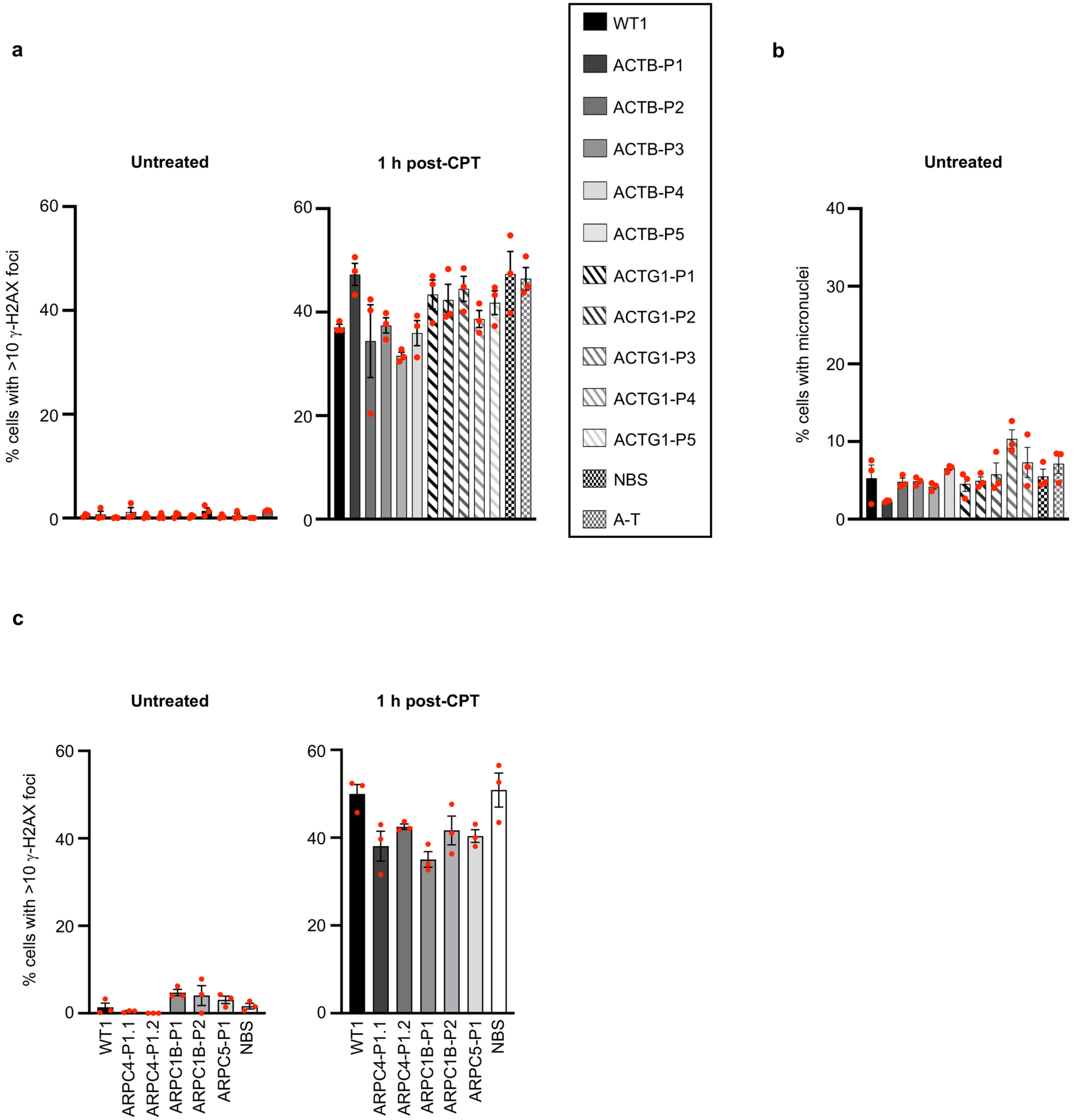

a
Untreated
1 h post-CPT
% cells with >10 γ-H2AX foci
60
40
20
0
WT1
ACTB-P1
ACTB-P2
ACTB-P3
ACTB-P4
ACTB-P5
ACTG1-P1
ACTG1-P2
ACTG1-P3
ACTG1-P4
ACTG1-P5
NBS
A-T
b
Untreated
% cells with micronuclei
40
30
20
10
0
c
Untreated
1 h post-CPT
% cells with >10 γ-H2AX foci
60
40
20
0
WT1
ARPC4-P1.1
ARPC4-P1.2
ARPC1B-P1
ARPC1B-P2
ARPC5-P1
NBS

Supplementary Fig. 11. Cells from BWCFF syndrome with mutations in *ACTG1* but not *ACTB* exhibit a DSB repair defect.

a-b Quantification of γ-H2AX foci (a) and micronuclei (b) in patient-derived fibroblasts with *de novo* mutations in *ACTB* or *ACTG1* before and after a 1 h exposure to 100 nM CPT. γ-H2AX foci and micronuclei were visualised by immunofluorescence microscopy and quantified in untreated cells and cells 1 h following the removal of the CPT. The mean of n=3 independent experiments is shown with the SEM. A minimum of 500 cells were counted per time point, per experiment. Statistical significance was calculated using unpaired Student's T-test (two-sided, equal variance). **c** Quantification of γ-H2AX foci in patient-derived fibroblasts with mutations in *ARPC1B*, *ARPC4* or *ARPC5* before and after a 1 h exposure to 100 nM CPT. γ-H2AX foci were visualised by immunofluorescence microscopy and quantified in untreated cells and cells 1 h following the removal of the CPT. The mean of n=3 independent experiments is shown with the SEM. A minimum of 500 cells were counted per time point, per experiment. Statistical significance was calculated using unpaired Student's T-test (two-sided, equal variance). Source data are provided as a Source Data file.

a

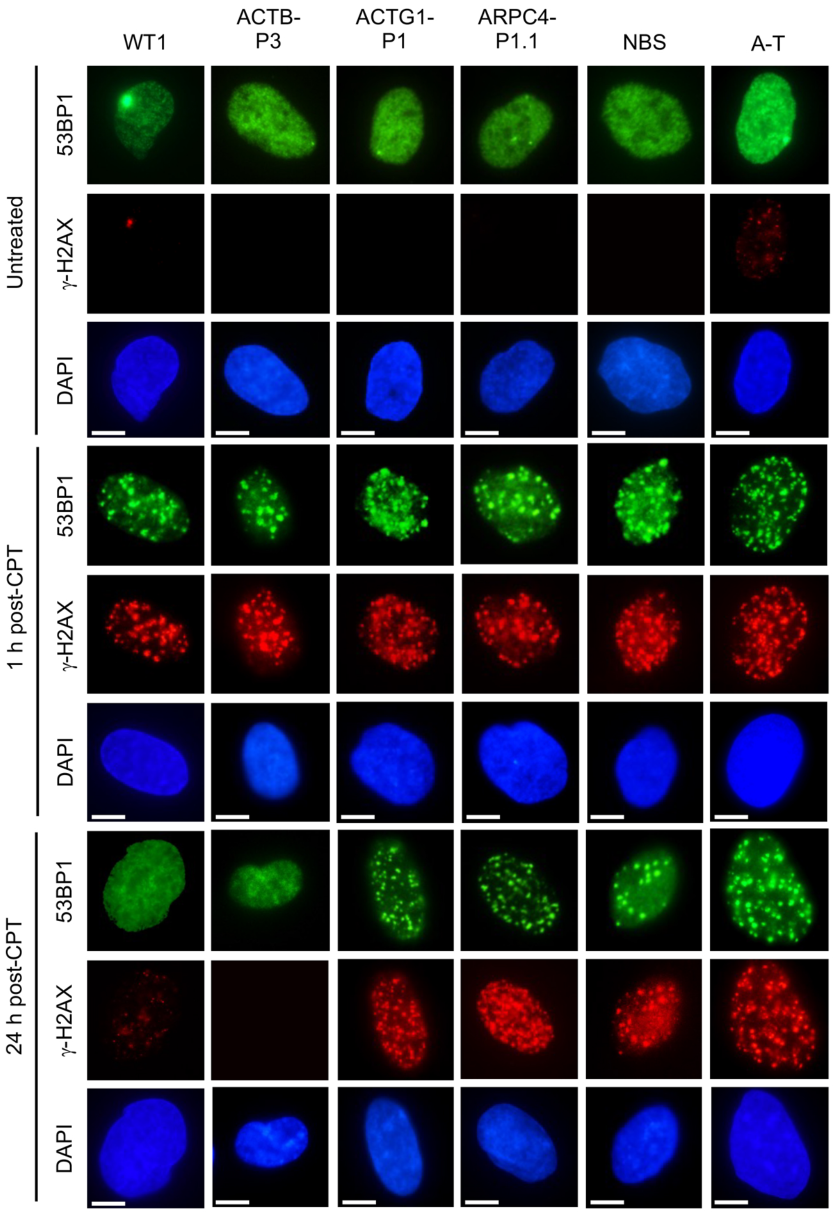

WT1
ACTB-
P3
ACTG1-
P1
ARPC4-
P1.1
NBS
A-T
Untreated
1 h post-CPT
24 h post-CPT
53BP1
γ-H2AX
DAPI

b

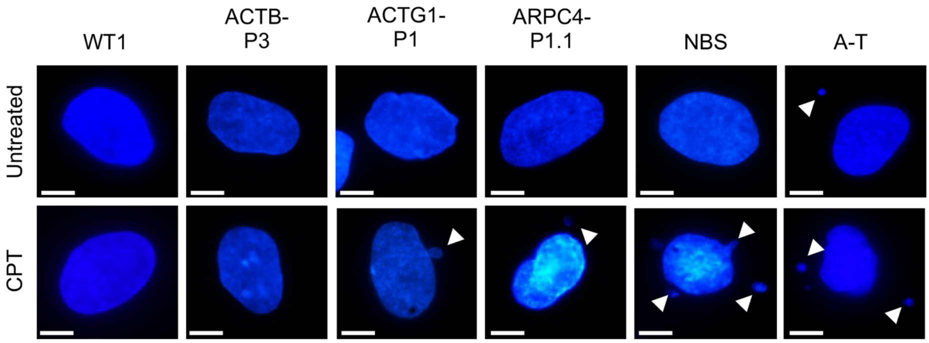

WT1
ACTB-
P3
ACTG1-
P1
ARPC4-
P1.1
NBS
A-T
Untreated
CPT

Supplementary Fig. 12. Cells from BWCFF syndrome with mutations in *ACTG1* but not *ACTB* exhibit a DSB repair defect.

a Representative images used for the quantification of CPT-induced γ-H2AX foci in patient-derived cell lines with mutations in *ACTB*, *ACTG1*, *ARPC1B*, *ARPC4* and *ARPC5* (Fig. 6b, 6f and Supplementary Fig. 11a and 11c). The scale bars represent 10 μm. **b** Representative images used for the quantification of CPT-induced micronuclei in patient-derived cell lines with mutations in *ACTB*, *ACTG1*, *ARPC1B*, *ARPC4* and *ARPC5* (Fig. 6c, 6g and Supplementary Fig. 11b). The scale bars represent 10 μm.

a

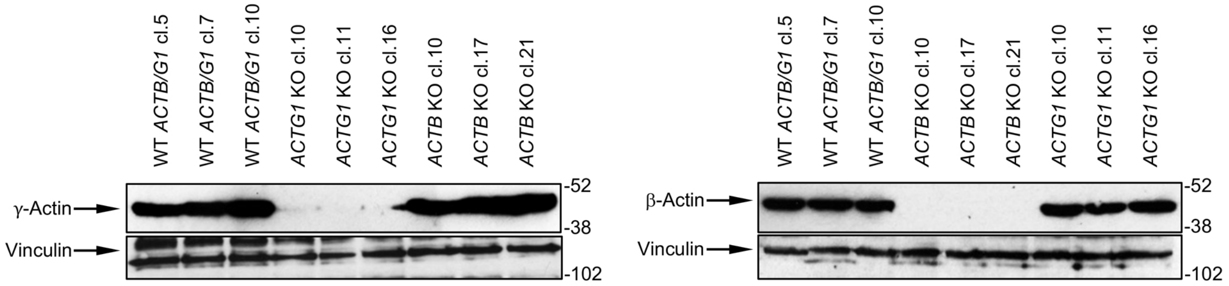
WT ACTB/G1 cl.5
WT ACTB/G1 cl.7
WT ACTB/G1 cl.10
ACTG1 KO cl.10
ACTG1 KO cl.11
ACTG1 KO cl.16
ACTB KO cl.10
ACTB KO cl.17
ACTB KO cl.21
γ-Actin
Vinculin
-52
-38
-102
WT ACTB/G1 cl.5
WT ACTB/G1 cl.7
WT ACTB/G1 cl.10
ACTB KO cl.10
ACTB KO cl.17
ACTB KO cl.21
ACTG1 KO cl.10
ACTG1 KO cl.11
ACTG1 KO cl.16
β-Actin
Vinculin
-52
-38
-102

b

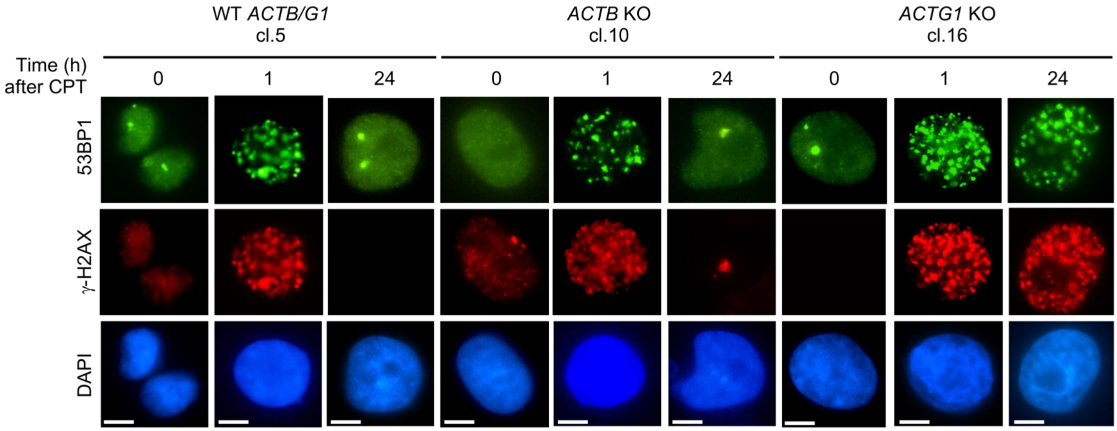
WT ACTB/G1
cl.5
ACTB KO
cl.10
ACTG1 KO
cl.16
Time (h)
after CPT
0
1
24
53BP1
γ-H2AX
DAPI

c

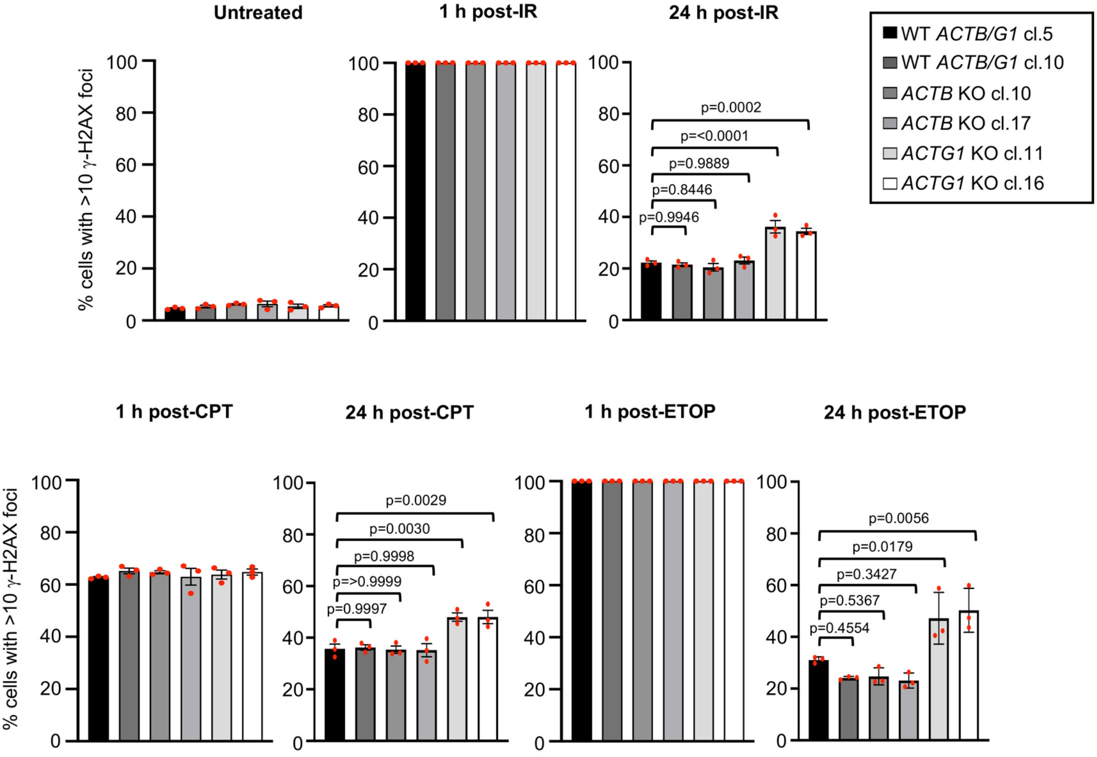
Untreated
1 h post-IR
24 h post-IR
WT ACTB/G1 cl.5
WT ACTB/G1 cl.10
ACTB KO cl.10
ACTB KO cl.17
ACTG1 KO cl.11
ACTG1 KO cl.16
% cells with >10 γ-H2AX foci
p=0.0002
p=<0.0001
p=0.9889
p=0.8446
p=0.9946
1 h post-CPT
24 h post-CPT
1 h post-ETOP
24 h post-ETOP
p=0.0029
p=0.0030
p=0.9998
p=>0.9999
p=0.9997
p=0.0056
p=0.0179
p=0.3427
p=0.5367
p=0.4554

Supplementary Fig. 13. A375 melanoma cells deficient in γ-actin but not β-actin exhibit a DSB repair defect.

a A Western blot showing the level of γ-actin and β-actin in three independent CRISPR knockout clones of *ACTB* and *ACTG1* in A375 cells relative to three independent WT *ACTB*/*ACTG1* A375 cell clones. n=1 independent experiments. **b-c** Quantification of γ-H2AX foci in two independent WT *ACTB*/*ACTG1* A375 clones, two independent *ACTB* knockout A375 clones and two independent *ACTG1* knockout A375 clones before and after exposure to 3 Gy IR, 100 nM CPT for 1 h or 10 μM ETOP for 30 min. γ-H2AX/53BP1 foci were visualised by immunofluorescence microscopy and quantified in untreated cells and cells 1 h and 24 h post-treatment. The mean of n=3 independent experiments is shown with the SEM. A minimum of 500 cells were counted per time point, per experiment. Statistical significance was calculated using: (c) an ordinary one-way ANOVA. Representative images of the cells at different time points before and after exposure to CPT are shown (b). The scale bars represent 10 μm. Source data are provided as a Source Data file.

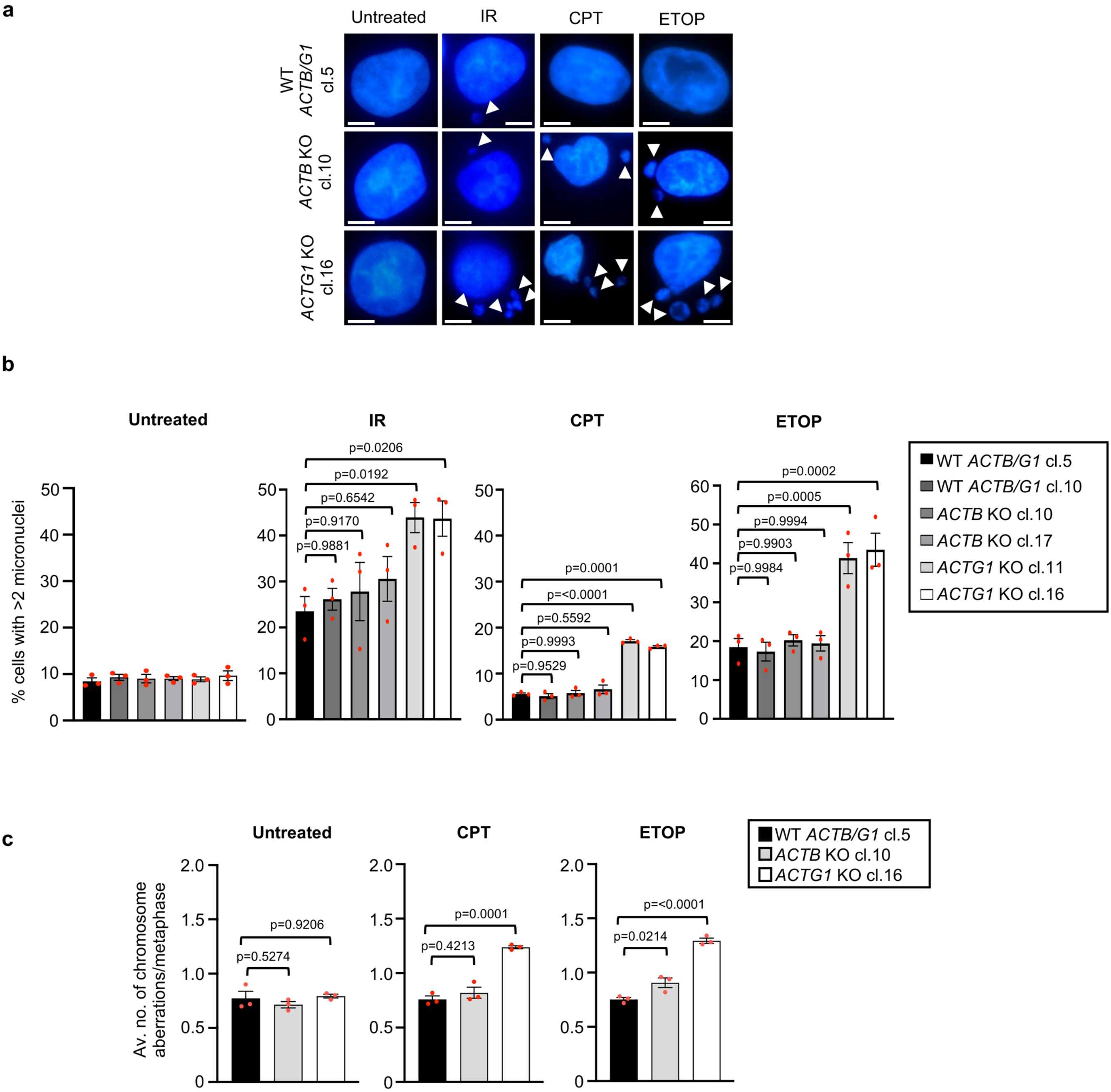
a
Untreated
IR
CPT
ETOP
WT ACTB/G1 cl.5
ACTB KO cl.10
ACTG1 KO cl.16
b
Untreated
IR
CPT
ETOP
% cells with >2 micronuclei
p=0.0206
p=0.0192
p=0.6542
p=0.9170
p=0.9881
p=0.0001
p=<0.0001
p=0.5592
p=0.9993
p=0.9529
p=0.0002
p=0.0005
p=0.9994
p=0.9903
p=0.9984
WT ACTB/G1 cl.5
WT ACTB/G1 cl.10
ACTB KO cl.10
ACTB KO cl.17
ACTG1 KO cl.11
ACTG1 KO cl.16
c
Untreated
CPT
ETOP
Av. no. of chromosome aberrations/metaphase
p=0.9206
p=0.5274
p=0.0001
p=0.4213
p=<0.0001
p=0.0214
WT ACTB/G1 cl.5
ACTB KO cl.10
ACTG1 KO cl.16

Supplementary Fig. 14. A375 melanoma cells deficient in γ-actin but not β-actin exhibit genome instability following exposure to IR, CPT and ETOP.

a-b Micronuclei were quantified from two independent clones of WT *ACTB/ACTG1* A375 cells, *ACTB* CRISPR knockout A375 cells and *ACTG1* CRISPR knockout A375 cells following exposure to 3 Gy IR, 100nM CPT for 1 h or 10 μM ETOP for 30 min. The mean of n=3 independent experiments is shown with the SEM. A minimum of 500 cells were counted per time point, per experiment. Representative images of the cells at different time points before and after treatment are shown (a). The scale bars represent 10 μm. **c** Quantification of chromosome aberrations in two independent clones of WT *ACTB/ACTG1* A375 cells, *ACTB* CRISPR knockout A375 cells and *ACTG1* CRISPR knockout A375 cells following chronic exposure to low dose CPT (5 nM) or ETOP (50 nM) for 24 h. Chromosome aberrations includes chromatid/chromosome gaps/breaks, chromatid/chromosome fragments and chromosome radials/exchanges. The mean of n=3 independent experiments is shown with the SEM. A minimum of 50 metaphases were counted per cell line in each experiment. Statistical significance was calculated using: (b &c) an ordinary one-way ANOVA. Source data are provided as a Source Data file.

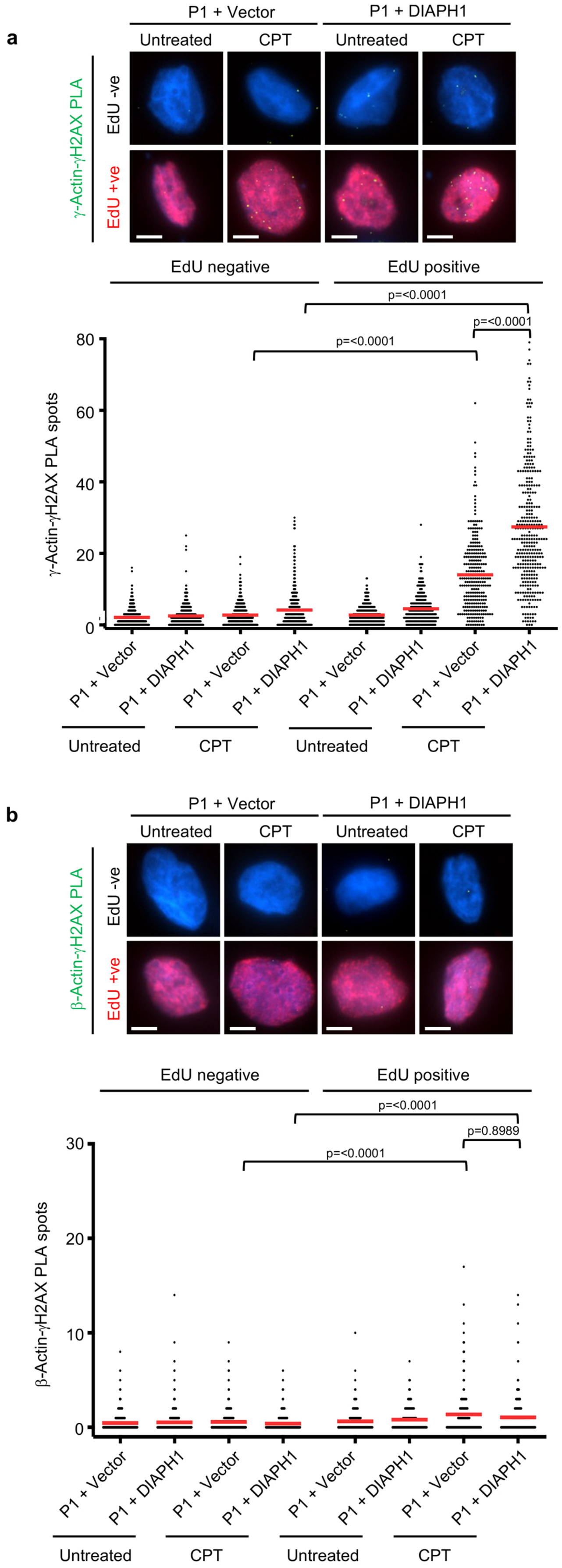
a
P1 + Vector
P1 + DIAPH1
Untreated
CPT
Untreated
CPT
γ-Actin-γH2AX PLA
EdU -ve
EdU +ve
EdU negative
EdU positive
p=<0.0001
p=<0.0001
p=<0.0001
γ-Actin-γH2AX PLA spots
80
60
40
20
0
P1 + Vector
P1 + DIAPH1
P1 + Vector
P1 + DIAPH1
P1 + Vector
P1 + DIAPH1
P1 + Vector
P1 + DIAPH1
Untreated
CPT
Untreated
CPT
b
P1 + Vector
P1 + DIAPH1
Untreated
CPT
Untreated
CPT
β-Actin-γH2AX PLA
EdU -ve
EdU +ve
EdU negative
EdU positive
p=<0.0001
p=0.8989
p=<0.0001
β-Actin-γH2AX PLA spots
30
20
10
0
P1 + Vector
P1 + DIAPH1
P1 + Vector
P1 + DIAPH1
P1 + Vector
P1 + DIAPH1
P1 + Vector
P1 + DIAPH1
Untreated
CPT
Untreated
CPT

Supplementary Fig. 15. γ-actin but not β-actin is localised to sites of DNA DSBs.

a-b Complemented DIAPH1-P1 fibroblasts were treated with 10 μM EdU for 30 min, exposed to 100 nM CPT for 1 h and then permeabilised/fixed. EdU positive cells were labelled with Alexa-fluor-488 using click chemistry. Cells were then subjected to a proximity ligation assay (PLA) reaction using antibodies to γH2AX and (a) γ-actin (b) or β-actin. (Top) Representative images of the PLA reaction (green spots) in EdU positive (red nuclei) and negative (blue) nuclei stained with DAPI. (Bottom) PLA spots were quantified in at least 50 EdU positive and 50 EdU negative cells per experiment per cell line per condition. The mean number of PLA spots per cell for n=3 independent experiments is shown (red line). Statistical significance was calculated using a Kruskal-Wallis test ([a] p=<0.0001, [b] p=<0.0001). Source data are provided as a Source Data file.

a

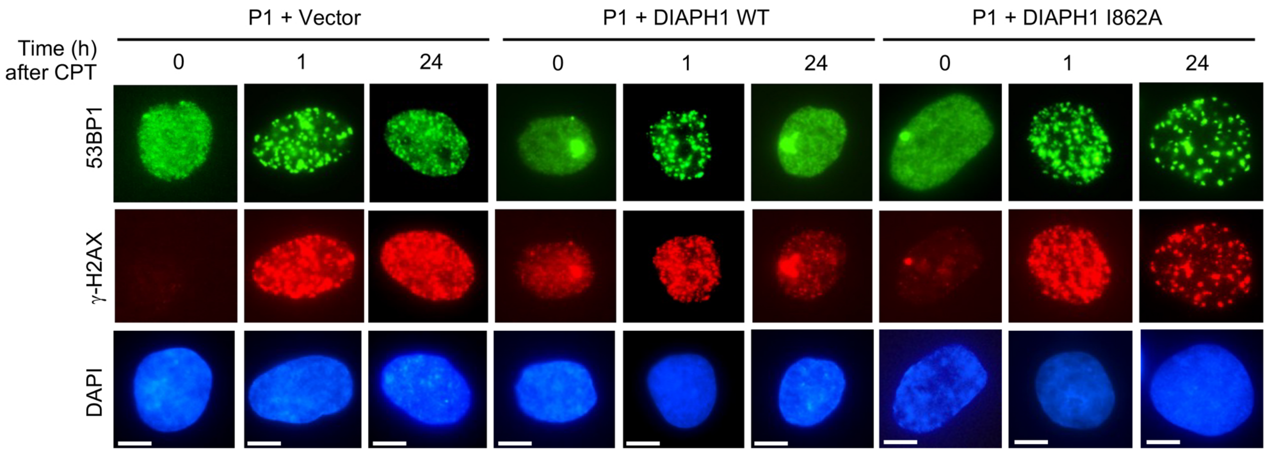

P1 + Vector
P1 + DIAPH1 WT
P1 + DIAPH1 I862A
Time (h) after CPT
0
1
24
0
1
24
0
1
24
53BP1
γ-H2AX
DAPI

b

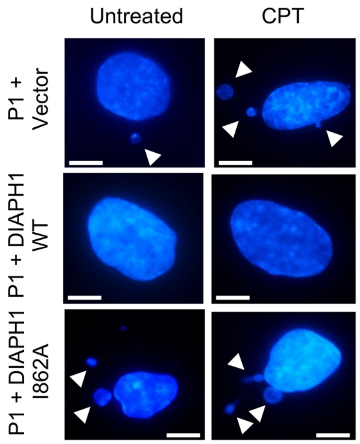

Untreated
CPT
P1 + Vector
P1 + DIAPH1 WT
P1 + DIAPH1 I862A

c

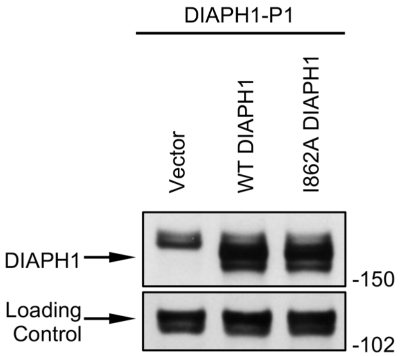

DIAPH1-P1
Vector
WT DIAPH1
I862A DIAPH1
DIAPH1
Loading Control
-150
-102

Supplementary Fig. 16. An actin nucleation deficient mutant of DIAPH1 is unable to support DNA DSB repair.

a-b Representative immunofluorescence images of DIAL syndrome patient P1-derived fibroblasts complemented with either an empty vector, WT DIAPH1 or a nucleation defective mutant (I862A) before and after a 1 h exposure to 100 nM CPT that were quantified in Fig. 6d-5e. The scale bars represent 10 μm. **c** Western blot showing the level of expression of WT and the I862A mutant DIAPH1 protein in complemented patient-P1 fibroblasts. A cross-reacting band is used as a control for protein loading. n=1 independent experiments. Source data are provided as a Source Data file.

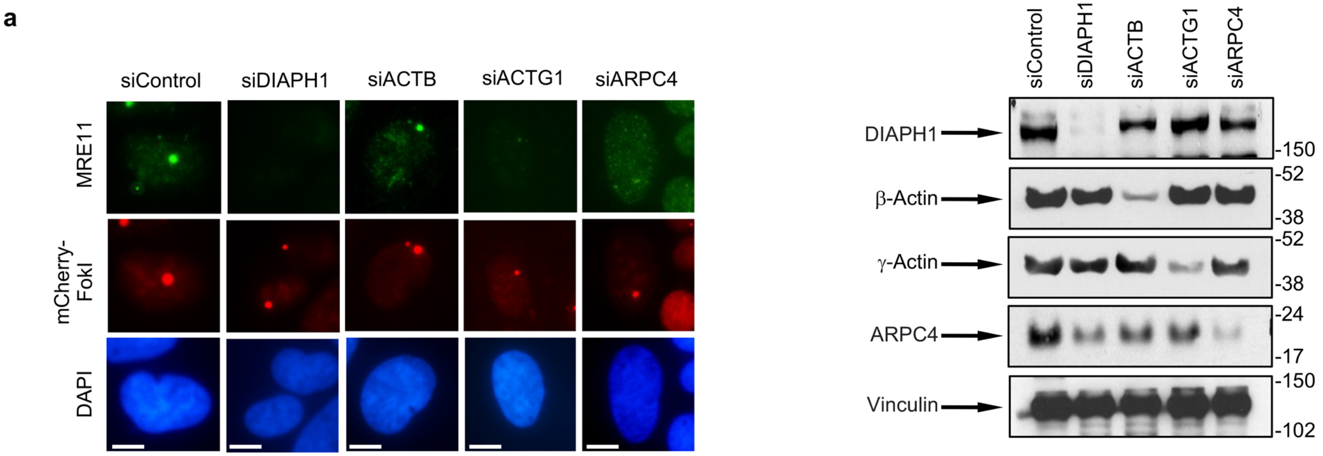
a
siControl
siDIAPH1
siACTB
siACTG1
siARPC4
MRE11
mCherry-FokI
DAPI
DIAPH1
β-Actin
γ-Actin
ARPC4
Vinculin
-150
-52
-38
-52
-38
-24
-17
-150
-102

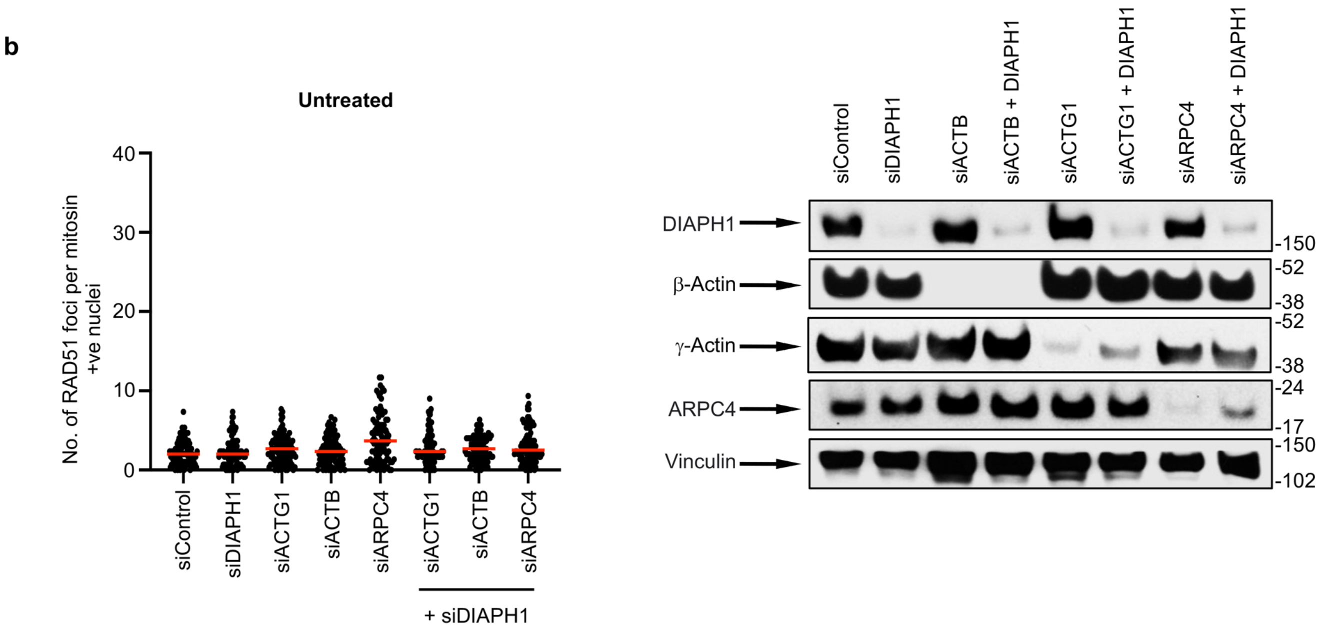
b
Untreated
No. of RAD51 foci per mitosin +ve nuclei
40
30
20
10
0
siControl
siDIAPH1
siACTG1
siACTB
siARPC4
siACTG1
siACTB
siARPC4
+ siDIAPH1
siControl
siDIAPH1
siACTB
siACTB + DIAPH1
siACTG1
siACTG1 + DIAPH1
siARPC4
siARPC4 + DIAPH1
DIAPH1
β-Actin
γ-Actin
ARPC4
Vinculin
-150
-52
-38
-52
-38
-24
-17
-150
-102

Supplementary Fig. 17. DIAPH1, γ-actin and ARPC4 regulate MRE11-dependent HR repair.

a (Left) Representative immunofluorescence images of MRE11 recruitment to FokI-induced DSBs quantified in Fig. 7b. The scale bars represent 10 μm. (Right) Western blotting on U-2 OS FokI-mCherry whole cell extracts showing the knockdown efficiency of the indicated siRNA used in Fig. 7b. Vinculin is used as a protein loading control. n=1 independent experiments. **b** (Left) HeLa cells were transfected with the indicated siRNAs for 18 h. 48 h post-transfection cells were treated with 100 nM CPT for 1h and then fixed/permeabilised 4 h post-CPT treatment. Cells were stained with antibodies to RAD51 and Mitosin (as a marker of S/G2 cells). RAD51 foci were quantified in a minimum of 100 S/G2 cells, per experiment. The mean of n=3 independent experiments with the SEM is shown (red line). Statistical significance was calculated using a Mann-Whitney rank sum test. (Right) Western blotting on HeLa whole cell extracts showing the knockdown efficiency of the indicated siRNA. Vinculin is used as a protein loading control. n=1 independent experiments. The same cells were used to quantify CPT-induced RAD51 foci in Fig. 7d. Source data are provided as a Source Data file.

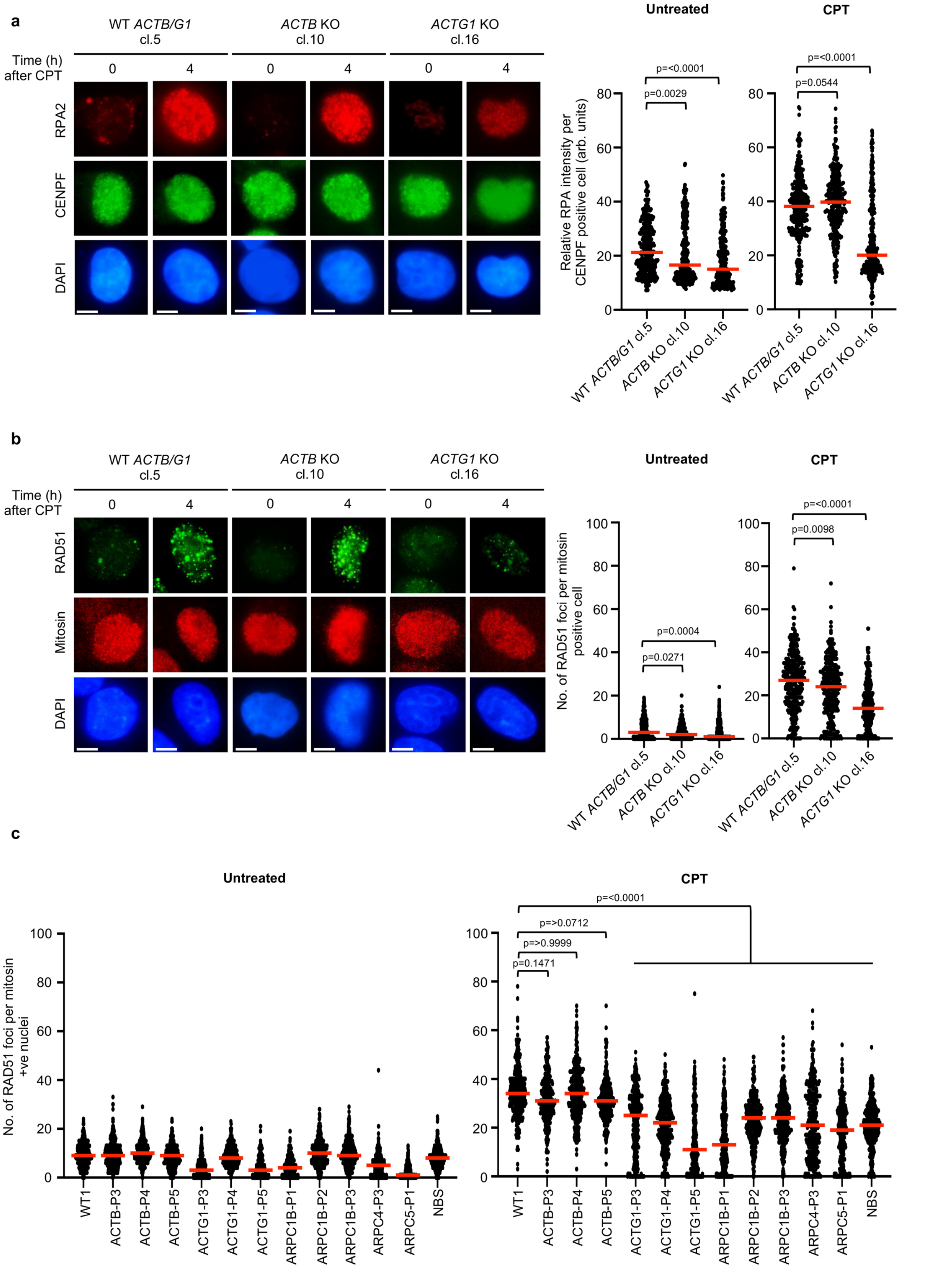

a
WT ACTB/G1 cl.5
ACTB KO cl.10
ACTG1 KO cl.16
Time (h) after CPT
0
4
RPA2
CENPF
DAPI
Untreated
CPT
p=<0.0001
p=0.0029
p=0.0544
Relative RPA intensity per CENPF positive cell (arb. units)
WT ACTB/G1 cl.5
ACTB KO cl.10
ACTG1 KO cl.16
b
RAD51
Mitosin
p=0.0004
p=0.0271
p=0.0098
No. of RAD51 foci per mitosin positive cell
c
No. of RAD51 foci per mitosin +ve nuclei
p=0.1471
p=>0.9999
p=>0.0712
WT1
ACTB-P3
ACTB-P4
ACTB-P5
ACTG1-P3
ACTG1-P4
ACTG1-P5
ARPC1B-P1
ARPC1B-P2
ARPC1B-P3
ARPC4-P3
ARPC5-P1
NBS

Supplementary Fig. 18. A375 melanoma cells deficient in γ-actin but not β-actin fail to efficiently relocalise RPA2 and RAD51 to sites of CPT-induced DNA damage.

a-b WT A375 cells or A375 cells knocked out for *ACTB* or *ACTG1* were exposed to 100 nM CPT for 1 h and then permeabilised/fixed 4 h following CPT removal. Cells were stained with an antibody to (a) RPA2 or (b)RAD51 in conjunction with an antibody to CENPF (as a marker of S2/G2 cells). RAD51 foci and RPA2 fluorescence intensity were quantified in a minimum of 100 cells, per experiment. The mean of n=3 independent experiments with the SEM is shown (red line). **c** Patient-derived cell lines with mutations in *ACTB*, *ACTG1*, *ARPC1B* and *ARPC5* were exposed to 100 nM CPT for 1 h and then fixed/permeabilised 4 h post-treatment. Cells were stained with antibodies to RAD51 and Mitosin (as a marker of S/G2 cells). RAD51 foci were quantified in a minimum of 100 S/G2 cells, per experiment. The mean of n=3 independent experiments with the SEM is shown (red line). Statistical significance was calculated using: (a-c) a Kruskal-Wallis test ([a-untreated] p=<0.001, [a-CPT] p=<0.0001, [b-untreated] p=0.0007, [b-CPT] p=<0.0001, [c-CPT] p=<0.0001). The scale bars represent 10 μm. Source data are provided as a Source Data file.

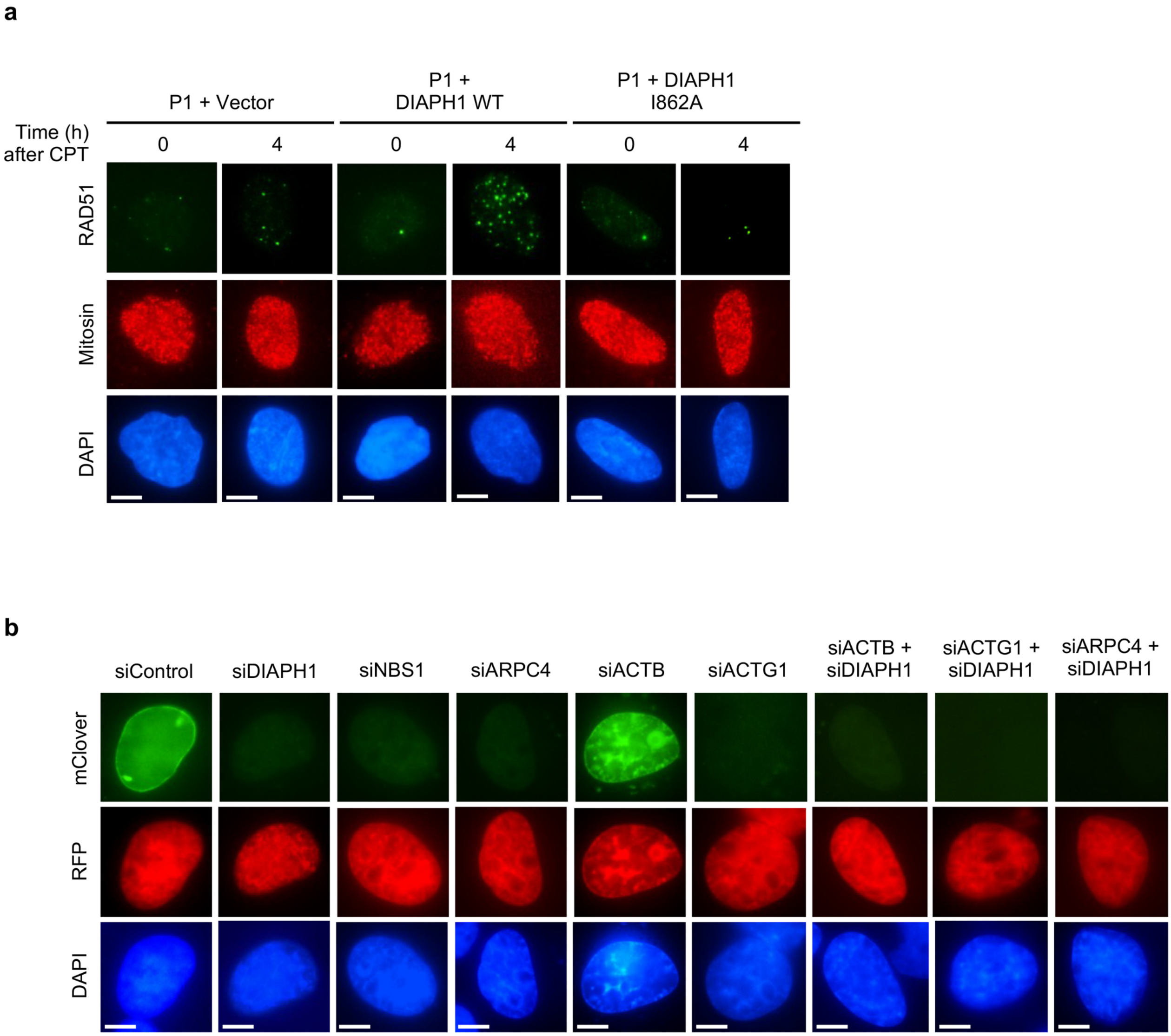
a
P1 + Vector
P1 + DIAPH1 WT
P1 + DIAPH1 I862A
Time (h) after CPT
0
4
0
4
0
4
RAD51
Mitosin
DAPI
b
siControl
siDIAPH1
siNBS1
siARPC4
siACTB
siACTG1
siACTB + siDIAPH1
siACTG1 + siDIAPH1
siARPC4 + siDIAPH1
mClover
RFP
DAPI

Supplementary Fig. 19. Depletion of DIAPH1, ARCP4 and γ-actin but not β-actin compromise the efficiency of HR-dependent DSB repair.

a Representative immunofluorescence images of DIAL syndrome patient P1-derived fibroblasts complemented with either an empty vector, WT DIAPH1 or a nucleation defective mutant (I862A) before and after a 4 h exposure to 100 nM CPT that were quantified in Fig. 7e. **b** Representative fluorescence images that were quantified in Fig. 7f as a measure of HR-dependent DSB repair in cells depleted of the indicated proteins. The scale bars represent 10 μm.

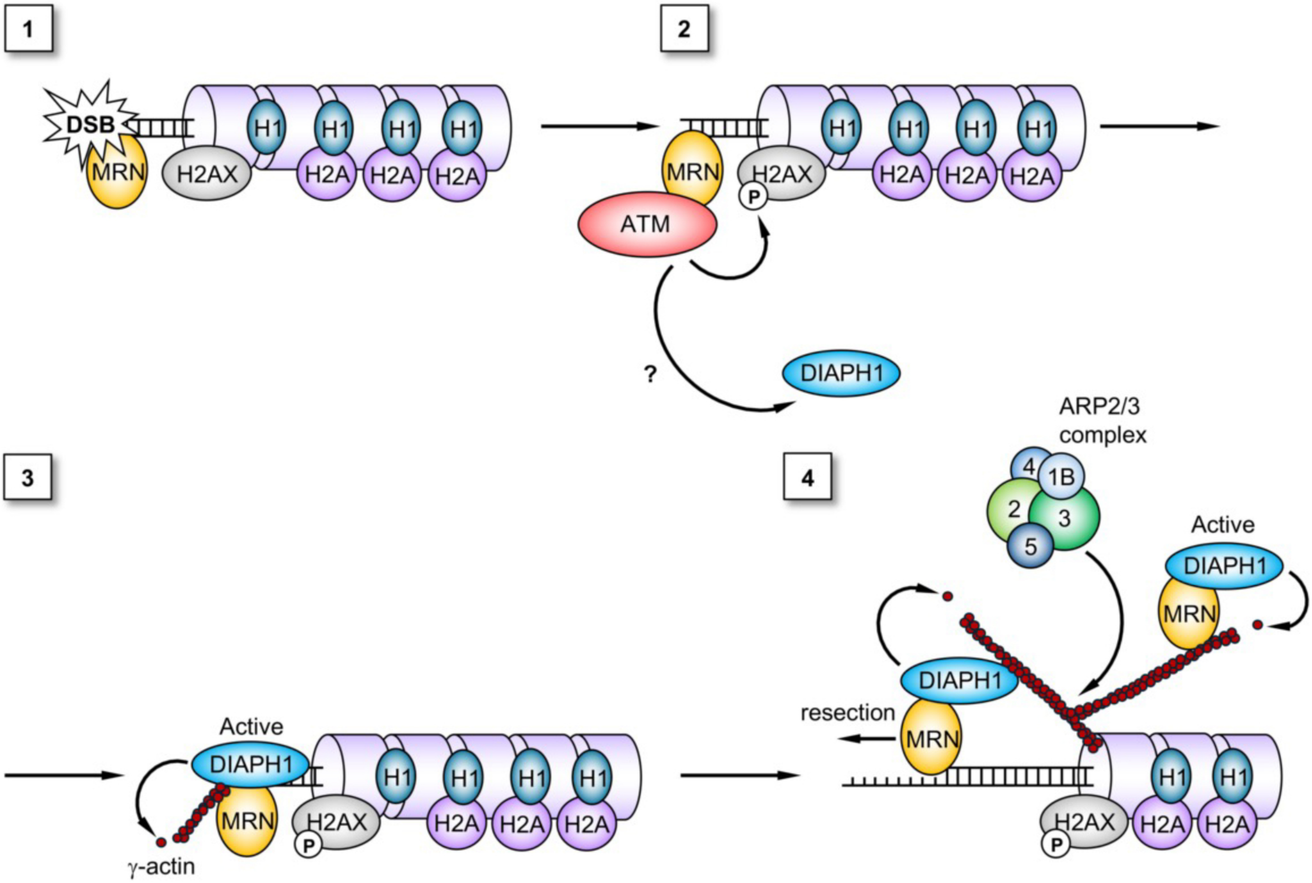
1
DSB
MRN
H2AX
H1
H2A
2
MRN
H2AX
P
ATM
?
DIAPH1
3
Active
DIAPH1
MRN
H2AX
P
γ-actin
4
ARP2/3
complex
4
1B
2
3
5
Active
DIAPH1
MRN
resection
H2AX
P
H1
H2A

Supplementary Fig. 20. A diagrammatic model of how DIAPH1 and γ-actin might function to regulate DSB repair.

1. The MRN (MRE11-RAD50-NBS1) complex binds to a DSB. **2.** The MRN complex recruits and activates ATM (Ataxia-Telangiectasia Mutated), which in turn phosphorylates histone H2AX (to form γ-H2AX). Additionally, ATM plays a role in promoting the recruitment of DIAPH1 to DNA DSBs, albeit the underlying mechanism is unclear. **3.** DIAPH1 binds to the MRN complex bound at DSBs and becomes activated. This triggers the polymerisation of linear γ-actin filaments within the vicinity of the DNA DSB. The red circles indicate globular actin that is nucleated to form a filament (F-actin). **4.** The localised linear γ-actin filament network functions to facilitate MRN-dependent DSB resection either by stabilising the MRN complex at DSBs undergoing processing or helping to further recruit MRN to damaged chromatin. These actin-dependent functions are facilitated by the ARP2/3 complex, which is involved in catalyzing the formation of branched actin filaments. It is possible that the formation of a localised branched actin filament network is critical for stabilising DSBs undergoing repair with the surrounding chromatin and/or enhances DNA repair protein localisation to a break by stimulating chromatin-repair protein liquid-liquid phase separation. H1, H2A and H2AX indicate histones H1, H2A and H2AX respectively. The ARP2/3 complex is compromised of the ARPC1B, ARPC2, ARPC3, ARPC4 and ARPC5 subunits, which are indicated as 1B 2, 3, 4 and 5 respectively. ‘P’ and ‘DSB’ indicate phosphorylation and DNA double strand break respectively. Light purple cylinders represent nucleosomes.

a

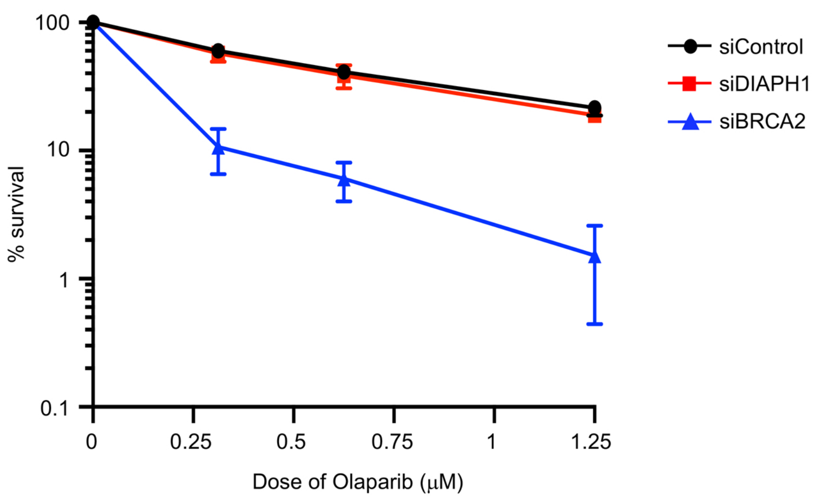

100
10
1
0.1
% survival
0
0.25
0.5
0.75
1
1.25
Dose of Olaparib (μM)
siControl
siDIAPH1
siBRCA2

b

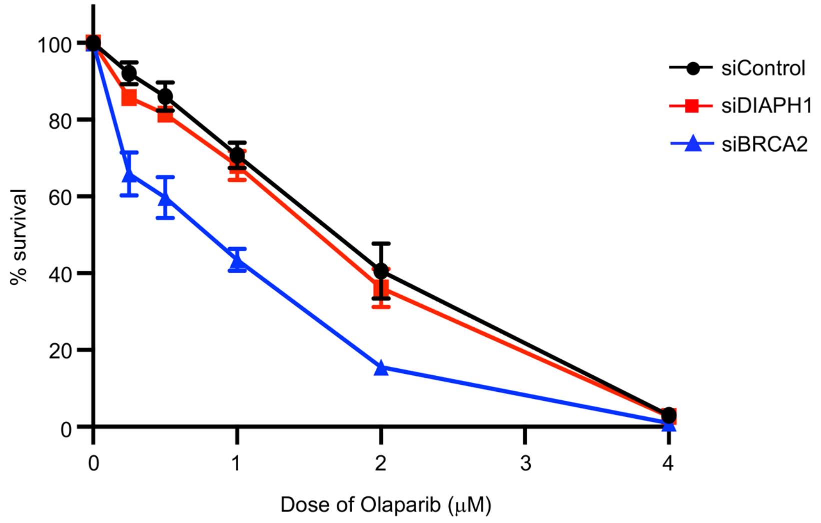

100
80
60
40
20
0
% survival
0
1
2
3
4
Dose of Olaparib (μM)
siControl
siDIAPH1
siBRCA2

Supplementary Fig. 21. Cells depleted of DIAPH1 do not display an increased sensitivity to Olaparib.

a-b U-2 OS cells (**a**) or Hela cells (**b**) transfected with either control, DIAPH1 or BRCA2 siRNA were plated at low density, exposed to continuous doses of Olaparib as indicated and were allowed to form colonies for 14 days. Colonies were fixed, stained and counted. Colonies were normalized to the number of cells plated and expressed as a mean % cell survival relative to the untreated control. Quantification of colonies from n=3 independent experiments. Source data are provided as a Source Data file.

Individual	Gene mutation	Inheritance	Clinical phenotype	Reference
ACTB-P1	c.474_475DupGG; p.Val159fs	*De novo*	Microcephaly, mild learning disability	Not published
ACTB-P2	c.587G>A; p.Arg196His	*De novo*	Short stature, moderate ID, epilepsy, muscular hypotonia, scoliosis, constipations, minor facial anomalies (Baraitser-Winter-Cerebrofrontalfacial syndrome)	Verloes et al.[19]
ACTB-P3	c.547C>T; p.Arg183Trp	*De novo*	Congenital bilateral deafness, generalized dystonia (dystonia-deafness syndrome)	Zech et al.[22]
ACTB-P4	c.547C>T; p.Arg183Trp	*De novo*	Congenital bilateral deafness, repeated ear infections, constipation (dystonia-deafness syndrome)	Not published
ACTB-P5	c.992_1008del; p.Ala331Valfs	*De novo*	Microcephaly, mild learning disability, thrombocytopenia	Latham et al.[21]
ACTB-P6	c.220G>A; p.Gly74Ser	*De novo*	Short stature, microcephaly, ID, pachygyria, cleft lip and palate, VSD, minor facial anomalies (Baraitser-Winter-Cerebrofrontalfacial syndrome)	Di Donato et al.[15] and Verloes et al.[19]
ACTG1-P1	c.608C>T; p.Thr203Met	*De novo*	Microcephaly, severe ID, epilepsy minor facial anomalies (Baraitser-Winter-Cerebrofrontalfacial syndrome)	Not published
ACTG1-P2	c.611C>G; p.Ala204Gly	*De novo*	Short stature, mild ID, ADHD, bilateral hearing loss, Intestinal pseudoobstruction at the neonatal period, TPN dependent, periodic tremors, progressive muscle atrophy	Not published
ACTG1-P3	c.548G>A; p.Arg183Gln	*De novo*	Progressive bilateral hearing loss, CI at 13y	Cabanillas et al.[20]
ACTG1-P4	c.221G>T; p.Gly74Val	*De novo*	Severe ID, severe microcephaly, agenesis of the corpus callosum, epilepsy, ptosis, renal duplication, liver cirrhosis, Hashimoto thyreoiditis, minor facial anomalies (Baraitser-Winter-Cerebrofrontalfacial syndrome)	Not published
ACTG1-P5	c.760C>T; p.Arg254Trp	*De novo*	Microcephaly, partial agenesis of the corpus callosum, dysgyria, epilepsy, hearing loss, sleep apnoe, minor facial anomalies (Baraitser-Winter-Cerebrofrontalfacial syndrome)	Not published
ARPC1B-P1	c.622G>T; p.Val208Phe (Hom)	Recessive	Cytopenia, thrombocytopenia, recurrent infections, hepatosplenomegaly, failure to thrive.	Brigida et al.[44]
ARPC1B-P2	c.212_226del; p.Gly71_Asn75del (Hom)	Recessive	Persistent eosinophilia, failure to thrive, recurrent infections, mild thrombocytopenia, lymphopenia.	Chiriaco et al.[45]
ARPC1B-P3	c.64+1G>A (Hom)	Recessive	Severe growth failure, eczema, lymphopenia, eosinophilia, thrombocytopenia, recurrent infections	Brigida et al.[44]
ARPC4-P1.1	c.472C>T; p.Arg158Cys	*De novo*	Microcephaly, mild motor delay, delayed speech, ADHD, facial telangiectasia, thumb-in-palm (splinted), strabismus.	Laboy Cintron et al.[46]
ARPC4-P1.2	c.472C>T; p.Arg158Cys	*De novo*	Microcephaly, mild motor delay, delayed speech, ADHD, facial telangiectasia, 5th finger camptodactyly.	Laboy Cintron et al.[46]
ARPC5-P4	c.23C>A; p.Ser8Ter (Hom)	Recessive	IUGR, short stature, facial dysmorphia, autoimmune haemolytic anaemia, thrombocytopenia, neutrophilia, recurrent infections, scoliosis, hepatosplenomegaly.	Nunes-Santos et al.[47]

Supplementary Table 1. Summary of the patient-derived cell lines with mutations in either *ACTB*, *ACTG1* or components of the ARP2/3 complex used.
