# Supplementary material for: Inherited deficiency of DIAPH1 identifies a DNA double strand break repair pathway regulated by γ-actin
Source: Nat Commun. 2025 May 14;16:4491. doi: 10.1038/s41467-025-59553-0 (PMC12078678; doi:10.1038/s41467-025-59553-0)
Supplement: Supplementary file 2 — Reporting Summary [file 41467_2025_59553_MOESM2_ESM.pdf]

nature portfolio

Corresponding author(s): Grant S. Stewart

Last updated by author(s): April 2025

Reporting Summary

Nature Portfolio wishes to improve the reproducibility of the work that we publish. This form provides structure for consistency and transparency in reporting. For further information on Nature Portfolio policies, see our Editorial Policies and the Editorial Policy Checklist.

Statistics

For all statistical analyses, confirm that the following items are present in the figure legend, table legend, main text, or Methods section.

n/a	Confirmed	
☐	☒	The exact sample size (n) for each experimental group/condition, given as a discrete number and unit of measurement
☒	☐	A statement on whether measurements were taken from distinct samples or whether the same sample was measured repeatedly
☐	☒	The statistical test(s) used AND whether they are one- or two-sided<br>*Only common tests should be described solely by name; describe more complex techniques in the Methods section.*
☒	☐	A description of all covariates tested
☒	☐	A description of any assumptions or corrections, such as tests of normality and adjustment for multiple comparisons
☐	☒	A full description of the statistical parameters including central tendency (e.g. means) or other basic estimates (e.g. regression coefficient) AND variation (e.g. standard deviation) or associated estimates of uncertainty (e.g. confidence intervals)
☒	☐	For null hypothesis testing, the test statistic (e.g. F, t, r) with confidence intervals, effect sizes, degrees of freedom and P value noted<br>*Give P values as exact values whenever suitable.*
☒	☐	For Bayesian analysis, information on the choice of priors and Markov chain Monte Carlo settings
☒	☐	For hierarchical and complex designs, identification of the appropriate level for tests and full reporting of outcomes
☒	☐	Estimates of effect sizes (e.g. Cohen's d, Pearson's r), indicating how they were calculated

Our web collection on statistics for biologists contains articles on many of the points above.

Software and code

Policy information about availability of computer code

Data collection	Super-resolution microscopy data were acquired using Micro-Manger (v 1.4).<br>Please see Methods section for detailed data acquisition description.
Data analysis	ImageJ v2.1.0, Matlab (R2020b).<br>Super-resolution image reconstruction were performed through C++ (via Intel Core i7 7800X) and CUDA8.0 (via NVIDIA GTX 1060) using the Maximum Likelihood Estimation (MLE) algorithm.<br>Please see Methods section for detailed data analysis description.<br>Codes for Auto- and Triple-Correlation algorithms, as well as a testing demo (with simulation codes) are available at https://github.com/yiny02/direct-Triple-Correlation-Algorithm. The code is for Research and Educational Purposes for Non-Profit Academic and/or Research Institutions.

For manuscripts utilizing custom algorithms or software that are central to the research but not yet described in published literature, software must be made available to editors and reviewers. We strongly encourage code deposition in a community repository (e.g. GitHub). See the Nature Portfolio guidelines for submitting code & software for further information.

Data

Policy information about availability of data

All manuscripts must include a data availability statement. This statement should provide the following information, where applicable:

- Accession codes, unique identifiers, or web links for publicly available datasets
- A description of any restrictions on data availability
- For clinical datasets or third party data, please ensure that the statement adheres to our policy

The datasets generated during WES are not publicly available due to reasons of sensitivity, e.g. human data, but may be available from the corresponding author upon request subject to parental consent. Gene variant frequency was obtained from the gnomAD database (https://gnomad.broadinstitute.org/). Accession codes for genes/proteins analysed within this study are: Human DIAPH1 (NM_005219). Plasmids obtained from Addgene (https://www.addgene.org/) used in this study: pLV-hTERT-IRES-hygro (Addgene #85140), psPax2 (Addgene #12260) and pMD2.G (Addgene #12259).

All unique materials, plasmids and cell cultures are freely available and can be obtained by contacting the corresponding authors. All imaging and single-molecule data constitute a sizable dataset (>10TB) that cannot be reasonably maintained online. Data will be made available by the corresponding authors upon reasonable request. Uncropped scans of all the Western blots presented within the manuscript are provided as a Source Data file. All raw data relating to the quantifications presented within the manuscript is provided as a Source Data file.

Field-specific reporting

Please select the one below that is the best fit for your research. If you are not sure, read the appropriate sections before making your selection.

☒ Life sciences ☐ Behavioural & social sciences ☐ Ecological, evolutionary & environmental sciences

For a reference copy of the document with all sections, see nature.com/documents/nr-reporting-summary-flat.pdf

Life sciences study design

All studies must disclose on these points even when the disclosure is negative.

Sample size	No statical tests were performed to determine sample size. Sample size for each experiment is indicated in the legend. Sample size was based on previous experimental experience using similar studies and assays employed within the field e.g. Abu-Libdeh et al (2022) J Clin Invest. 132:e147301; Bayley et al (2022) Mol Cell. 82:1924-1939; Higgs et al (2018) Mol Cell. 71:25-41; Reynolds et al (2017) Nature Genet. 49:537-549; Higgs et al (2015) Mol Cell. 59:462-77. The sample size of each experiment, such as how many cells were analyzed for each independent biological replicate, is indicated in the relevant Figure Legends.
Data exclusions	For all STORM imaging experiments, EDU-positive (as pre-established S-phase markers) nuclei were selected for analysis, which were then analyzed equally. Details of nucleus selection were described in the manuscript.<br>No other data exclusion were performed.
Replication	Each experiment was replicated, with the number of independent biological replicates (at least 3) being stated in the relevant Figure Legends.<br>All SR experiments were performed at least in triplicate with >50 sample size.<br>We followed the same protocols to generate replicates for each of our experiments, and the analysis of the data were reliably reproduced.
Randomization	All samples used in this study, including cultured cells, were allocated randomly to each condition. For all imaging experiments, nuclei on coverslips were randomly selected for imaging.
Blinding	Samples in each experiment to be analyzed by microscopy were randomized so the investigator was counting the experiment blind. Samples for gene sequencing, immunoblotting, immunoprecipitation, or samples that required the addition of different genotoxic compounds were known to the experimenter when preparing samples. In most cases where the identity of each sample was known to the experimenter was due to the fact that much of the data presented within the manuscript was provided back to the referring clinician as part of a report that was feedback to the affected family/patient about our work confirming the pathogenicity of the gene variants identified by whole exome sequencing. This is essential to reduce the possibility of mixing up patient samples or patient results that could compromise the clinical report.

Reporting for specific materials, systems and methods

We require information from authors about some types of materials, experimental systems and methods used in many studies. Here, indicate whether each material, system or method listed is relevant to your study. If you are not sure if a list item applies to your research, read the appropriate section before selecting a response.

Materials & experimental systems

n/a	Involved in the study	
☐	☒	Antibodies
☐	☒	Eukaryotic cell lines
☒	☐	Palaeontology and archaeology
☒	☐	Animals and other organisms
☐	☒	Human research participants
☐	☒	Clinical data
☒	☐	Dual use research of concern

Methods

n/a	Involved in the study	
☒	☐	ChIP-seq
☒	☐	Flow cytometry
☒	☐	MRI-based neuroimaging

Antibodies

Antibodies used

Antibodies used for Western blotting: pS1981 ATM (AF1655, 1:500) from R&D systems; Arp2 (sc-166103, 1:500) and U1-70K (sc-390988, 1:2000) from Santa Cruz Biotechnology; ATM (A300-299A, 1:500), pS824-KAP1 (A300-767A, 1:1000), KAP1 (A300-274A, 1:3000), pS966-SMC1 (A300-050A, 1:1000), SMC1 (A300-055A, 1:1000), Chk2 (A300-681A, 1:1000) and DIAPH1 (A300-077A, 1:500) from Fortis Life Sciences; pT68-CHK2 (2197, 1:500) from Cell Signalling Technology; H2A (07-146, 1:1000); γ-H2AX (05-636, 1:3000), beta-actin (A5316, 1:10000) and HA (H9658, 1:3000) from Sigma-Aldrich; pS343-NBS1 (ab47272, 1:500), Mre11 (ab214, 1:2000), Histone H3 (ab1791, 1:2000) and ARPC4 (ab217065, 1:1000) from Abcam; Nbs1 (GTX70224, 1:10000) from GeneTex; BRCA2 (OP95, 1:500), RPA2 (NA18, 1:1000), gamma-actin (MABT824, 1:10000) and Rad51 (PC130, 1:500) from Merck; Vinculin (66305-1, 1:5000) from Proteintech.

Antibodies used for immunofluorescence: γ-H2AX (Sigma-Aldrich, 05-636, 1:1000), Rad51 (Sigma-Aldrich, PC130, 1:500), 53BP1 (Novus-Biologicals, NB100-904, 1:1000), RPA2 (Abcam, ab2174, 1:250), BrdU (Abcam, BU1/75 (ICR1), 1:100), Mre11 (Abcam, ab214, 1:1000), BRCA1 (Santa Cruz Biotechnology, sc-6954, 1:200), CENPF (Antibody Atlas, HPA052382, 1:1000), Mitosin (BD Transduction labs, 610768, 1:1000), DIAPH1 (Fortis Life Sciences, A300-077A, 1:250).

Antibodies for STORM: Antibodies used for SMLM imaging: MRE11 [12D7] AF488 conjugated NB100473 (Novus), RPA70 [EPR3472] AF647 conjugated ab199240 (Abcam), BRCA1 [RAY] AF488 conjugated NB100598 (Novus).

Antibodies for PLA: Antibodies used for PLA: DIAPH1 (Fortis Life Sciences, A300-077A, 1:1000), γ-H2AX (Sigma-Aldrich, 05-636, 1:2000).

Validation

All commercially available antibodies were used as per the manufacturer's guidelines and used for the techniques in which they have been validated by the manufacturers.

We have previously validated these antibodies: anti-ATM, anti-phospho-ATM, anti-Chk1, anti-phospho-Chk1, anti-Nbs1, anti-phospho-Nbs1, anti-SMC1, anti-phospho-SMC1, anti-KAP1, anti-phospho-KAP1, anti-Chk2, anti-phospho-Chk2, anti-H2A, anti-γH2AX, anti-53BP1, anti-Rad51, anti-CENPF/Mitosin, anti-HA, anti-BRCA2, anti-Mre11 and anti-BrdU antibodies, for Western blotting and immunofluorescence in the following publications:

Grange LJ, Reynolds JJ, Ullah F, Isidor B, Shearer RF, Latypova X, Baxley RM, Oliver AW, Ganesh AN, Cooke SL, Jhujh SS, McNee GS, Hollingworth R, Higgs MR, Natsume T, Khan T, Martos-Moreno GÁ, Chupp S, Mathew CG, Parry D, Simpson MA, Nahavandi N, Yüksel Z, Drasdo M, Kron A, Vogt P, Jonasson A, Seth SA, Gonzaga-Jauregui C, Brigatti KW, Stegmann APA, Kanemaki M, Josifova D, Uchiyama Y, Oh Y, Morimoto A, Osaka H, Ammous Z, Argente J, Matsumoto N, Stumpel CTRM, Taylor AMR, Jackson AP, Bielinsky A-K, Mailand N, Le Caignec C, Davis EE, Stewart GS. (2022). Pathogenic variants in SLF2 and SMC5 cause segmented chromosomes and mosaic variegated hyperploidy. Nature Commun. 13:66644

Abu-Libdeh B, Jhujh SS, Dhar S, Sommers JA, Datta A, Longo GMC, Grange LJ, Reynolds JJ, Cooke SL, McNee GS, Hollingworth R, Woodward BL, Ganesh AN, Smerdon SJ, Nicolae CM, Durlacher-Betzer K, Molho-Pessach V, Abu-Libdeh A, Meiner V, Moldovan G-L, Roukos V, Harel T, Brosh Jr. RM, Stewart GS. (2022). RECON Syndrome is a genome instability disorder caused by mutations in the DNA helicase RECQL1. J Clin Invest. 132:e147301

Bayley R, Borel V, Moss RJ, Sweatman E, Ruis P, Ormrod A, Goula A, Mottram RMA, Stanage T, Hewitt G, Saponaro M, Stewart GS, Boulton SJ, Higgs MR. (2022). H3K4 methylation by SETD1A/BOD1L facilitates RIF1-dependent NHEJ. Mol Cell. 82:1924-1939

Higgs MR, Sato K, Reynolds JJ, Begum S, Bayley R, Goula A, Vernet A, Paquin KL, Skalnik DG, Kobayashi W, Takata M, Howlett NG, Kurumizaka H, Kimura H, Stewart GS. (2018). Histone methylation by SETD1A protects nascent DNA through the nucleosome chaperone activity of FANCD2. Mol Cell. 71:25-41

Reynolds JJ, Bicknell LS, Carroll P, Higgs MR, Shaheen R, Murray JE, Papadopoulos DK, Leitch A, Murina O, Tarnauskaitė Ž, Wessel SR, Zlatanou A, Vernet A, Kriegsheim A, Mottram RMA, Logan CV, Bye H, Li Y, Brean A, Maddirevula S, Challis RC, Skouloudaki K, Almoisheer A, Alsaif HS, Amar A, Prescott NJ, Bober MB, Duker A, Faqeih E, Seidahmed MZ, Tala SA, Alswaid A, Ahmed S, Al-Aama JY, Altmüller J, Balwi MA, Brady AF, Chessa L, Cox H, Fischetto R, Heller R, Henderson BD, Hobson E, Nürnberg P, Percin EF, Peron S, Spaccini L, Quigley AJ, Thakur S, Wise CA, Yoon G, Alnemer M, Tomancak P, Yigit G, Taylor AMR, Reijns MAM, Simpson MA, Cortez D, Alkuraya FS, Mathew CG, Jackson AP, Stewart GS. (2017). Mutations in DONSON disrupt replication fork stability and cause microcephalic dwarfism. Nature Genet. 49:537-549

Higgs MR, Reynolds JJ, Winczura A, Blackford AN, Borel V, Miller ES, Zlatanou A, Nieminuszczy J, Ryan EL, Davies NJ, Stankovic T, Boulton SJ, Niedzwiedz W, Stewart GS. (2015). BOD1L Is Required to Suppress Deleterious Resection of Stressed Replication Forks. Mol Cell. 59:462-77.

The anti-DIAPH1, anti-gamma-actin and anti-beta-actin were validated by Western blotting on cell extracts from knockout cell lines.

Eukaryotic cell lines

Policy information about cell lines

Cell line source(s)	Patient-derived lymphoblastoid cell lines (LCLs) were generated from peripheral blood samples with Epstein Barr virus (EBV) transformation using standard methods. Dermal primary fibroblasts were grown from skin-punch biopsies and were immortalized with a lentivirus expressing human telomerase reverse transcriptase (hTERT). The biological sex of the patients in our study was not considered as a relevant variable. However, the patient cohort studied contained 8 affected female patients and 2 affected male patients. U-2 OS and HeLa cells were obtained from ATCC (U-2 OS: HTB-96; HeLa: CCL-2) and 293FT cells were purchased from Invitrogen (293FT: R70007). Beta-actin and gamma-actin Knockout A375 cell lines were obtained from Antonina J. Mazur (Malek N, et al. Knockout of ACTB and ACTG1 with CRISPR/Cas9(D10A) technique shows that non-muscle beta and gamma actin are not equal in relation to human melanoma cells' motility and focal adhesion formation. Int J Mol Sci. 2020;21:2746). All BCWFF syndrome cell lines were obtained from Nataliya Di Donato (Verloes A, et al. Baraitser-Winter cerebrofrontalfacial syndrome: delineation of the spectrum in 42 cases. Eur J Hum Genet. 2015;23:292-301). Cell lines with mutations in ARPC1B, ARPC4 and ARPC5 were obtained from Heather C Mefford, Sergio D Rosenzweig, Stefano Volpi, Gigliola Di Matteo and Caterina Cancrini (Chiriaco M, et al. Radiosensitivity in patients affected by ARPC1B deficiency: a new disease trait? Front Immunol. 2022;13:919237; Laboy Cintron D, et al. A recurrent, de novo pathogenic variant in ARPC4 disrupts actin filament formation and causes microcephaly and speech delay. HGG Adv. 2021;3(1):100072; Nunes-Santos, CJ et al. Inherited ARPC5 mutations cause an actinopathy impairing cell motility and disrupting cytokine signalling. Nature Comms. 2023;14(1):3708). The ER-mCherry LacI-FokI-DD expressing U-2 OS cells were provided by Roger Greenberg (Shanbhag NM, et al. ATM-dependent chromatin changes silence transcription in cis to DNA double-strand breaks. Cell. 2010;141(6):970-981). The DR-GFP U-2 OS cells were provided by Jeremy Stark (Gunn A, Stark JM. I-Sce-I-based assays to examine distinct repair outcomes of mammalian chromosomal double strand breaks. Methods Mol Biol. 2012;920:379-391). The U-2 OS AsiSI-ER cells were provided by Gaelle Legube (Zhou Y, et al. Quantitation of DNA double-strand break resection intermediates in human cells. Nucleic Acids Res. 2014;42(3):e19).
Authentication	All patient derived cell lines were authenticated by sequencing and verifying the presence of the relevant mutations. 293FT, HeLa and U-2 OS cell lines were verified by STR profiling. The A375 beta and gamma-actin CRISPR knockout cells were verified by Western blotting.
Mycoplasma contamination	All cell lines were routinely tested for, and confirmed to be negative for, mycoplasma contamination.
Commonly misidentified lines (See ICLAC register)	No commonly misidentified cell lines were used in this study.

Human research participants

Policy information about studies involving human research participants

Population characteristics	The characteristics and clinical phenotypes of patients with mutations in DIAPH1 are included in Figure 1.
Recruitment	Patients with microcephaly and short stature who possessed mutations in DIAPH1 were identified by individual groups/ clinicians using whole exome sequencing, and the collaboration to study the pathological significance of the identified DIAPH1 was established via GeneMatcher. Patients were only selected to be recruited into the study if they were identified to have inherited biallelic, potentially pathogenic variants in the DIAPH1 gene that were inherited from both parents and segregated with the disease. Any patients with variants in DIAPH1 that were demonstrated not to be pathogenic (i.e. the variants did not affect mRNA expression, gene splicing, protein stability, protein localisation) or where re-expression of the WT gene in patient-derived cell lines did not complement any identified cellular defects, were excluded from the study. Patients with mono-allelic or de novo variants in DIAPH1 were excluded from the study due to difficulties with definitively demonstrating the pathogenicity of these variants in a small patient cohort. Patient derived cell lines with pathogenic variants in either ACTB, ACTG1, ARPC1B, ARPC4 or ARPC5 were identified from publications and were obtained from individual clinicians/researchers.
Ethics oversight	Informed consent was obtained from all participating families to take clinical samples and to publish clinical information in accordance with local approval regulations. This study was approved by the West Midlands, Coventry and Warwickshire Research Ethics Committee (REC: 20/WM/0098)

Note that full information on the approval of the study protocol must also be provided in the manuscript.

Clinical data

Policy information about clinical studies

All manuscripts should comply with the ICMJE guidelines for publication of clinical research and a completed CONSORT checklist must be included with all submissions.

Clinical trial registration	*Provide the trial registration number from ClinicalTrials.gov or an equivalent agency.*
Study protocol	*Note where the full trial protocol can be accessed OR if not available, explain why.*
Data collection	*Describe the settings and locales of data collection, noting the time periods of recruitment and data collection.*

Outcomes

Describe how you pre-defined primary and secondary outcome measures and how you assessed these measures.
