# Supplementary material for: Inherited deficiency of DIAPH1 identifies a DNA double strand break repair pathway regulated by γ-actin
Source: Nat Commun. 2025 May 14;16:4491. doi: 10.1038/s41467-025-59553-0 (PMC12078678; doi:10.1038/s41467-025-59553-0)

Figure 1

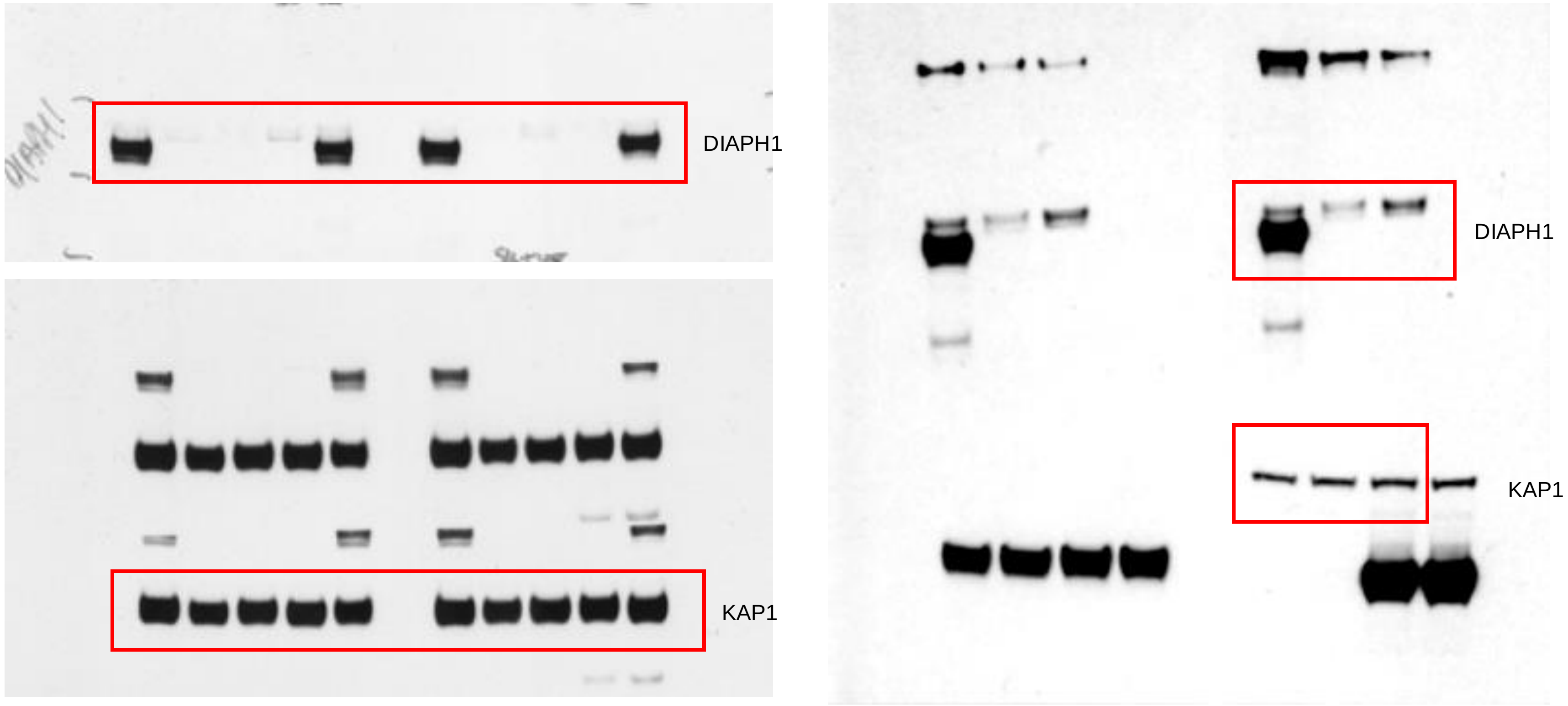

Figure 2

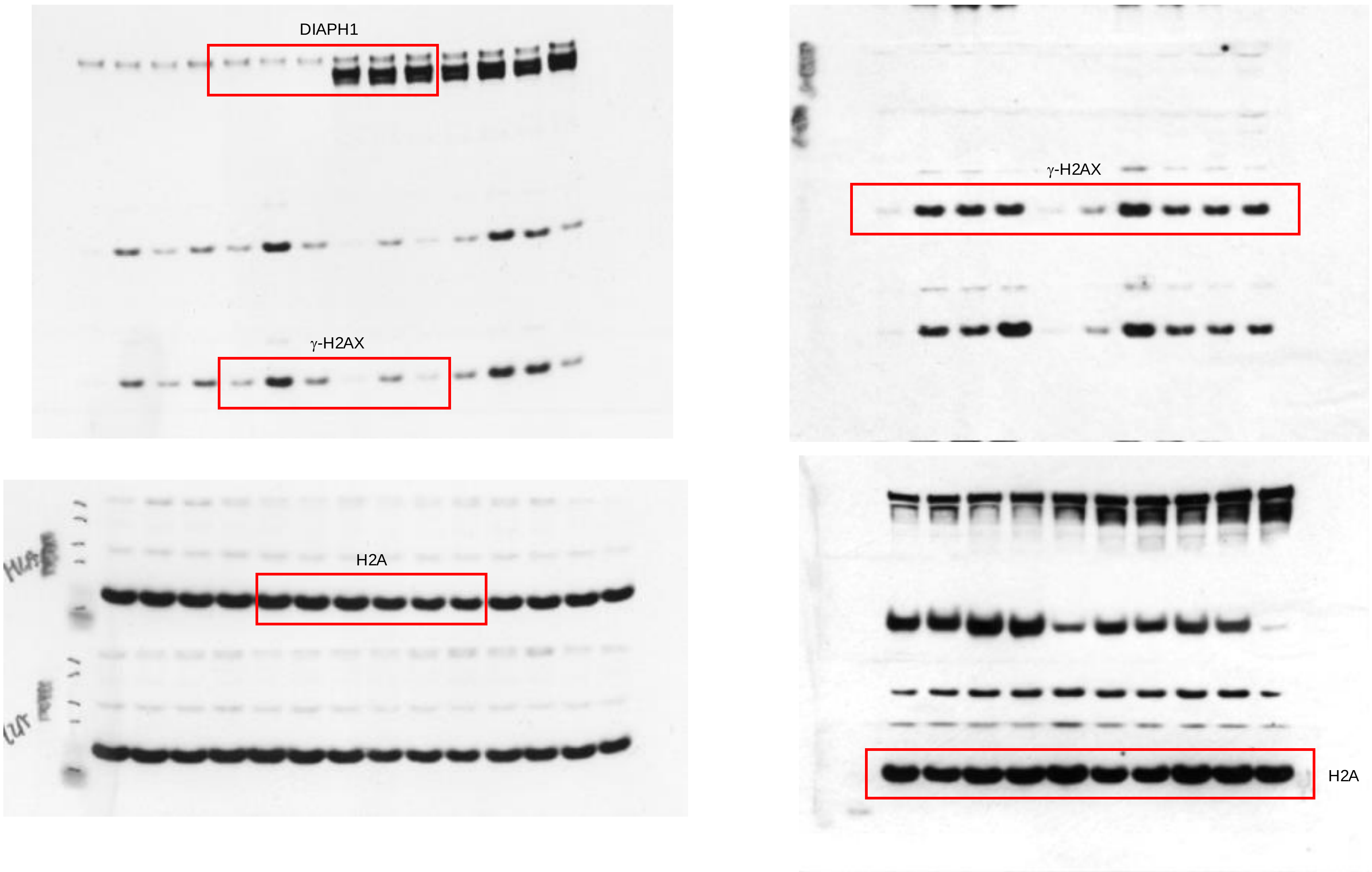

Figure 5C

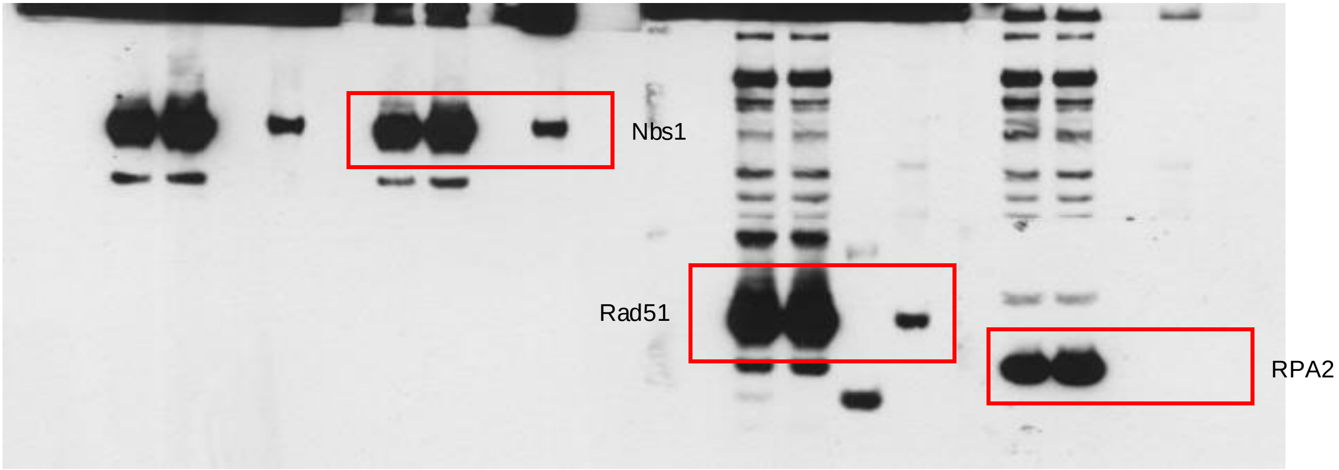

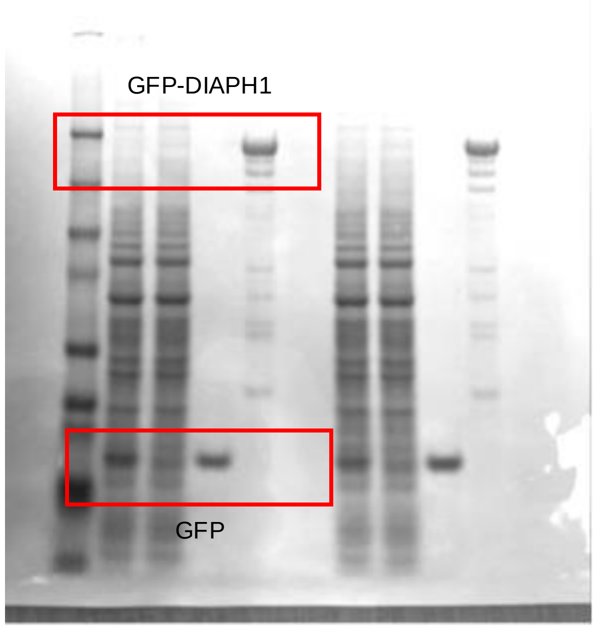

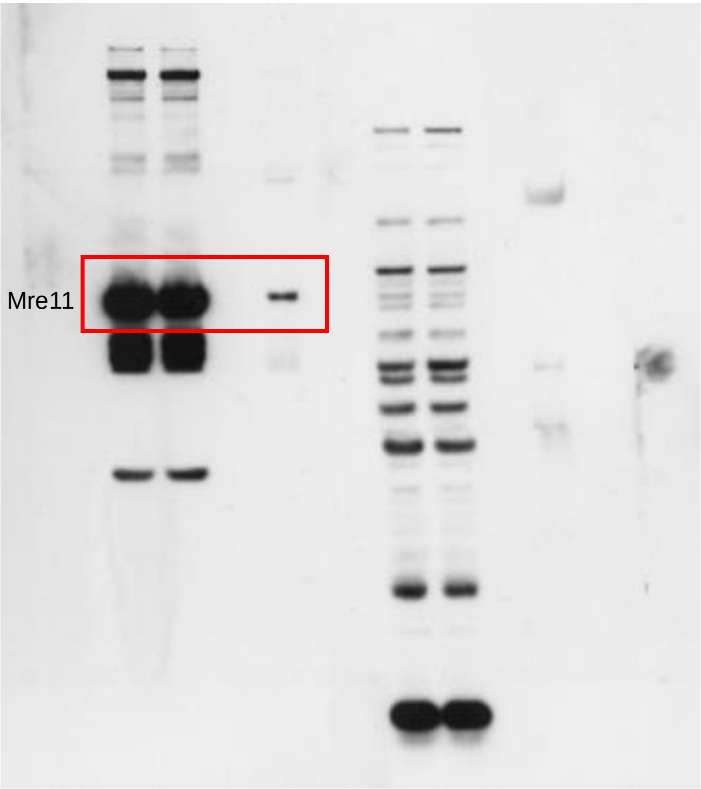

Figure 7A

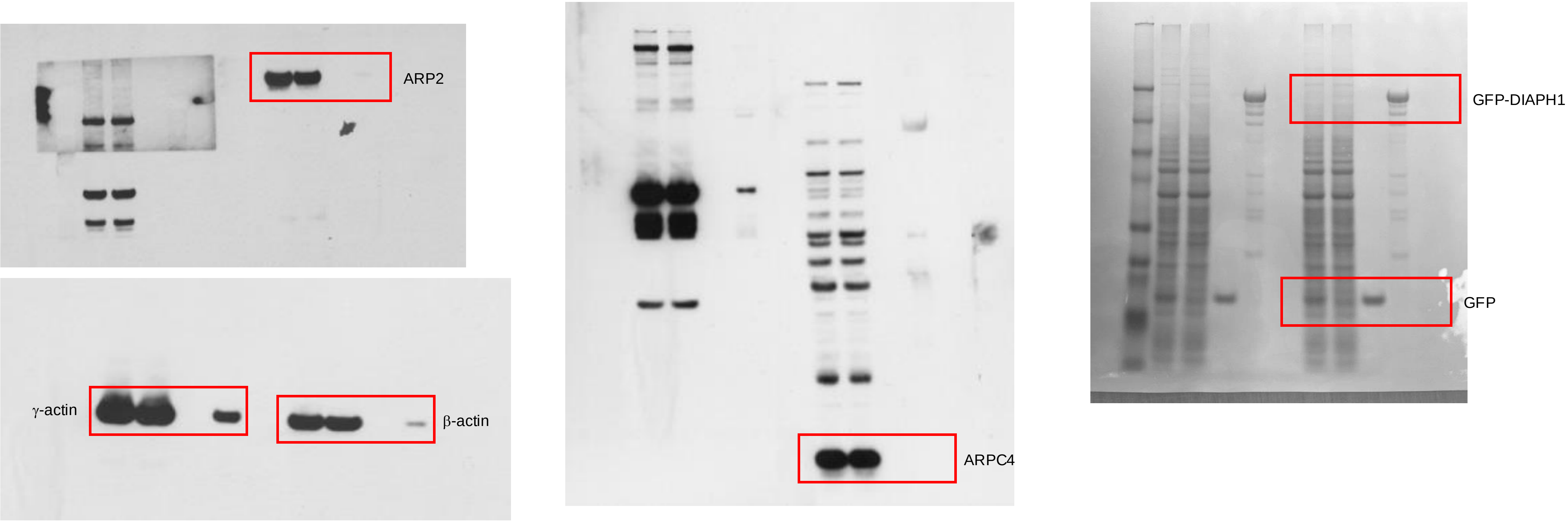

Figure S1

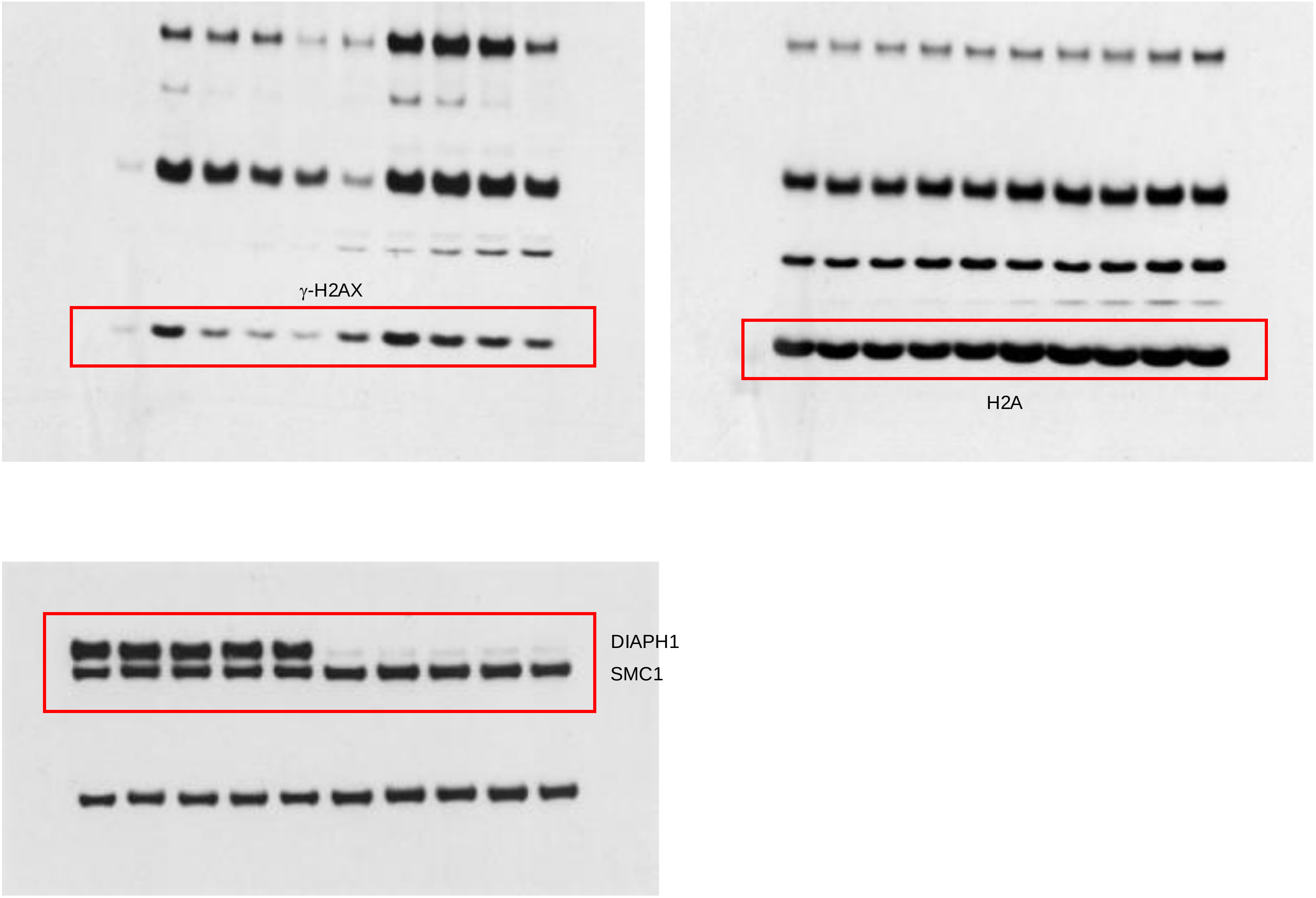

Figure S2A

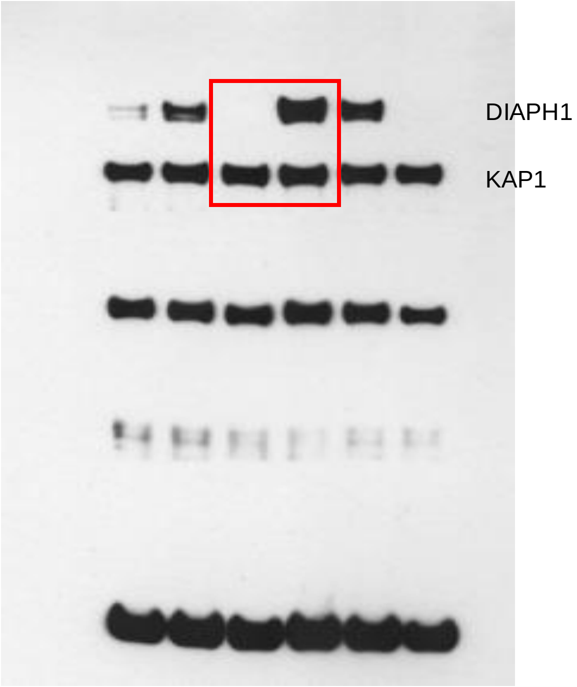

Figure S3A

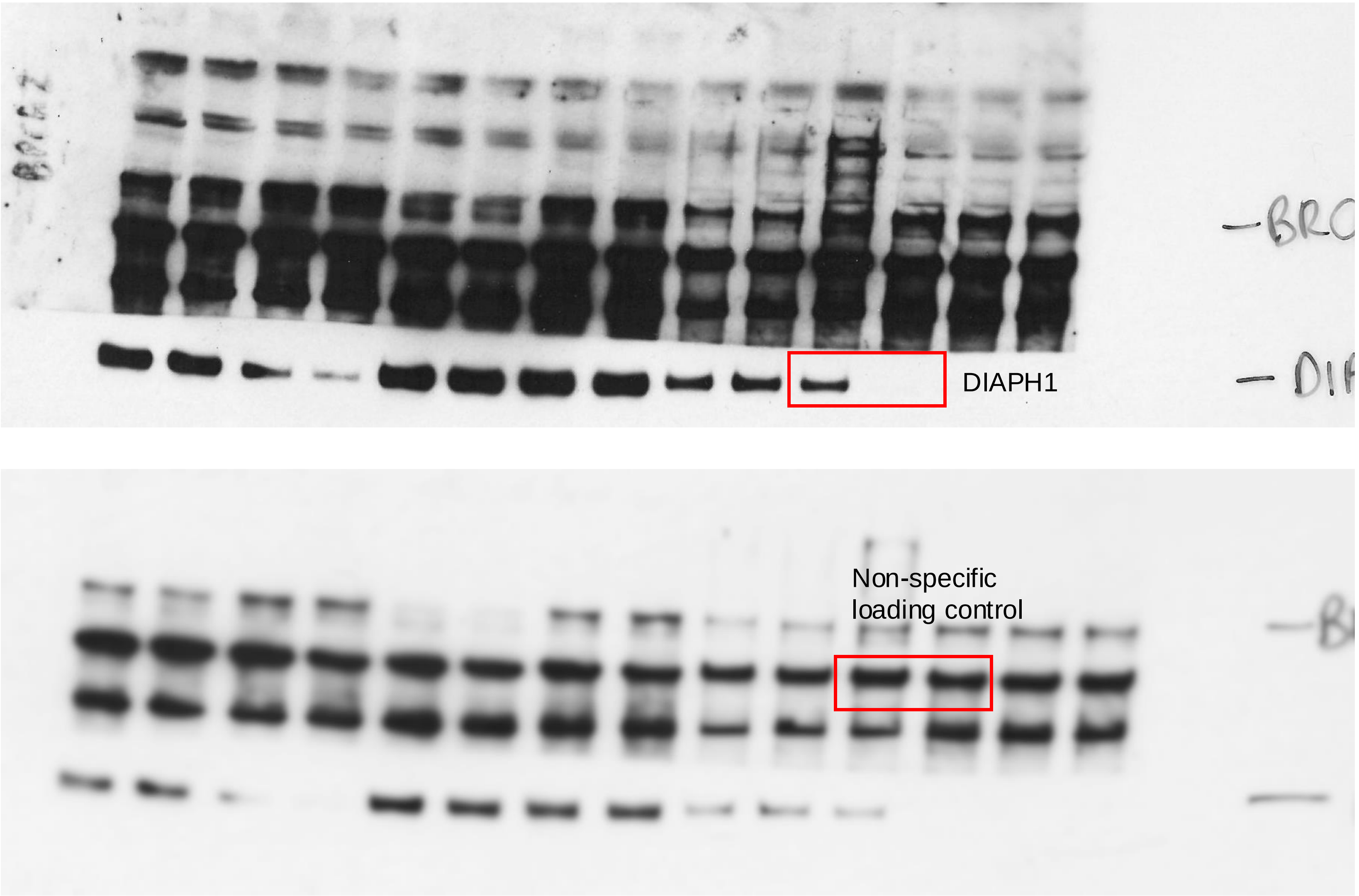

Figure S5C

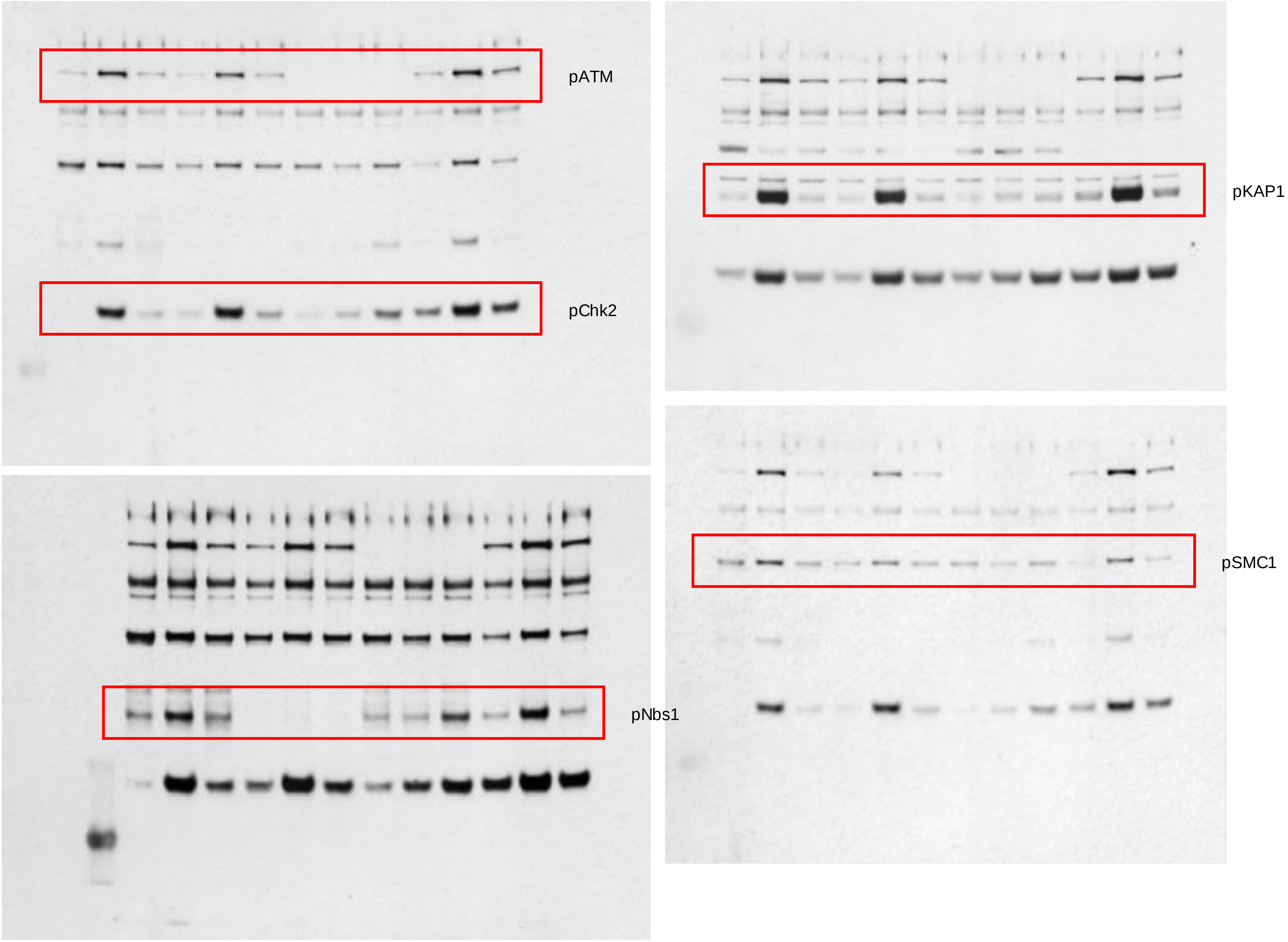

Figure S5C

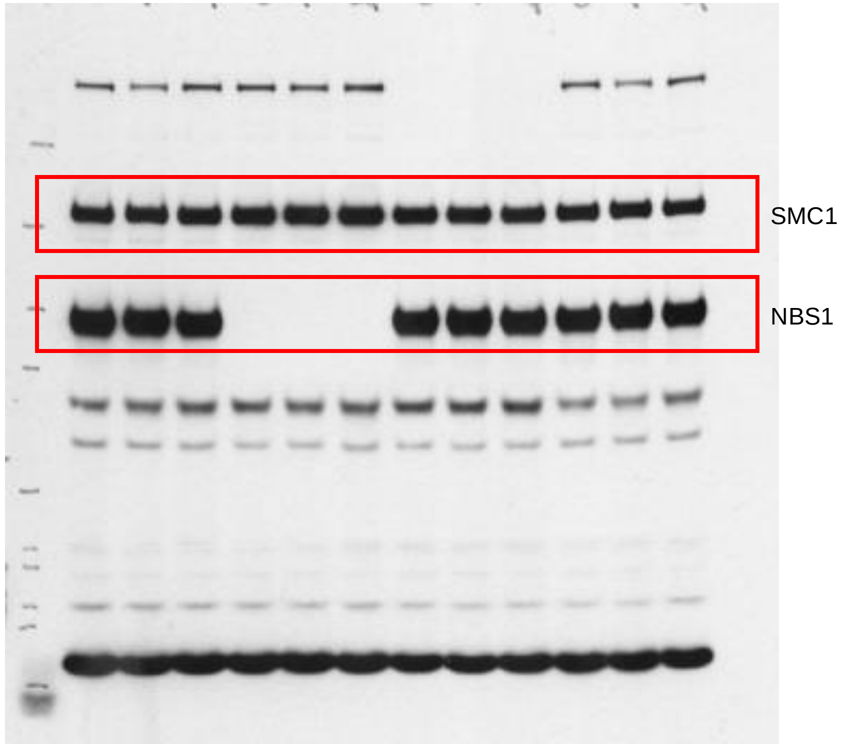

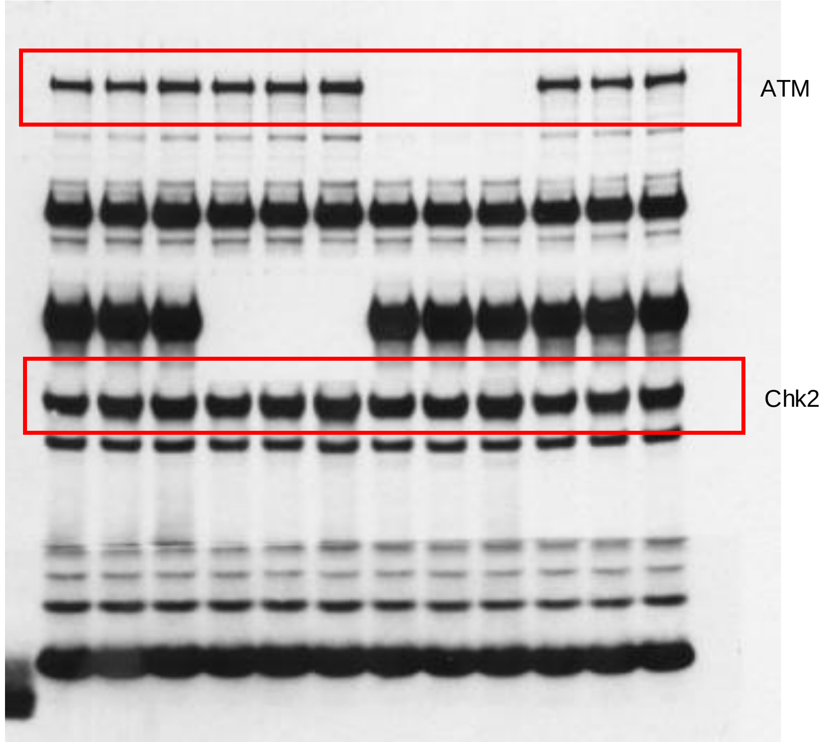

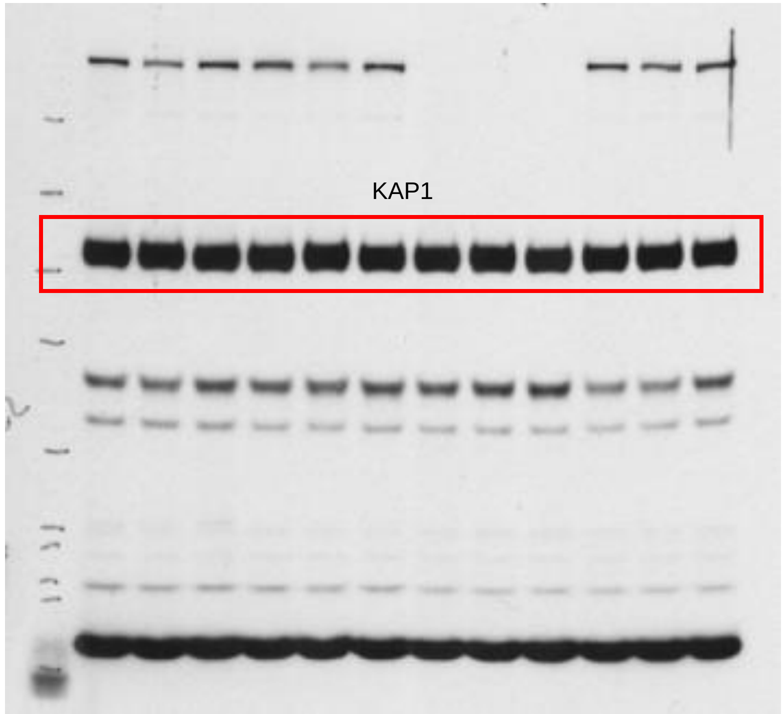

Figure S5E

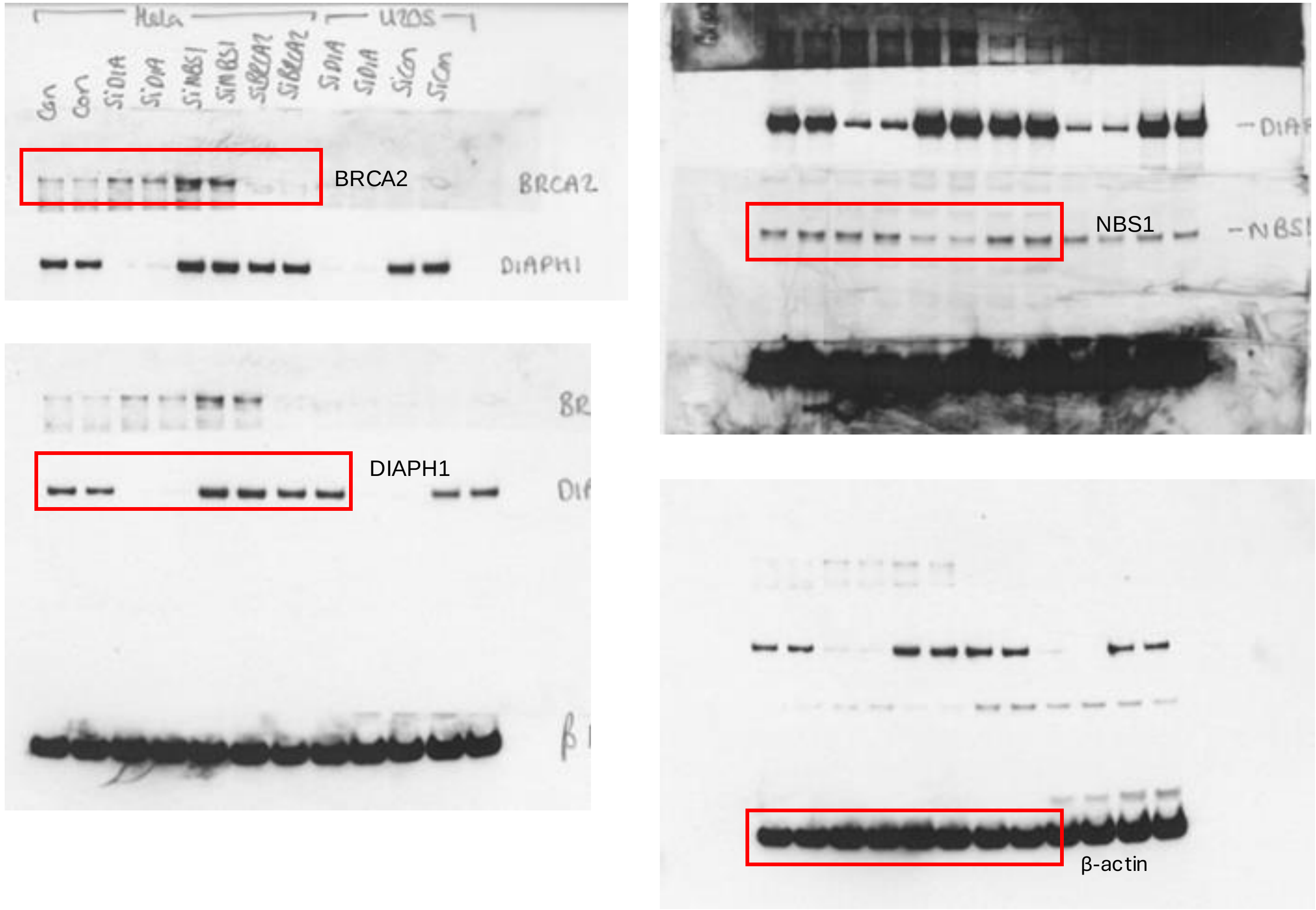

For consistency with how the data is presented, the siNBS1 and siBRCA2 cell lines were swapped around by flipping the blot, so the final figure appears they were loaded in a different order

Figure S8a

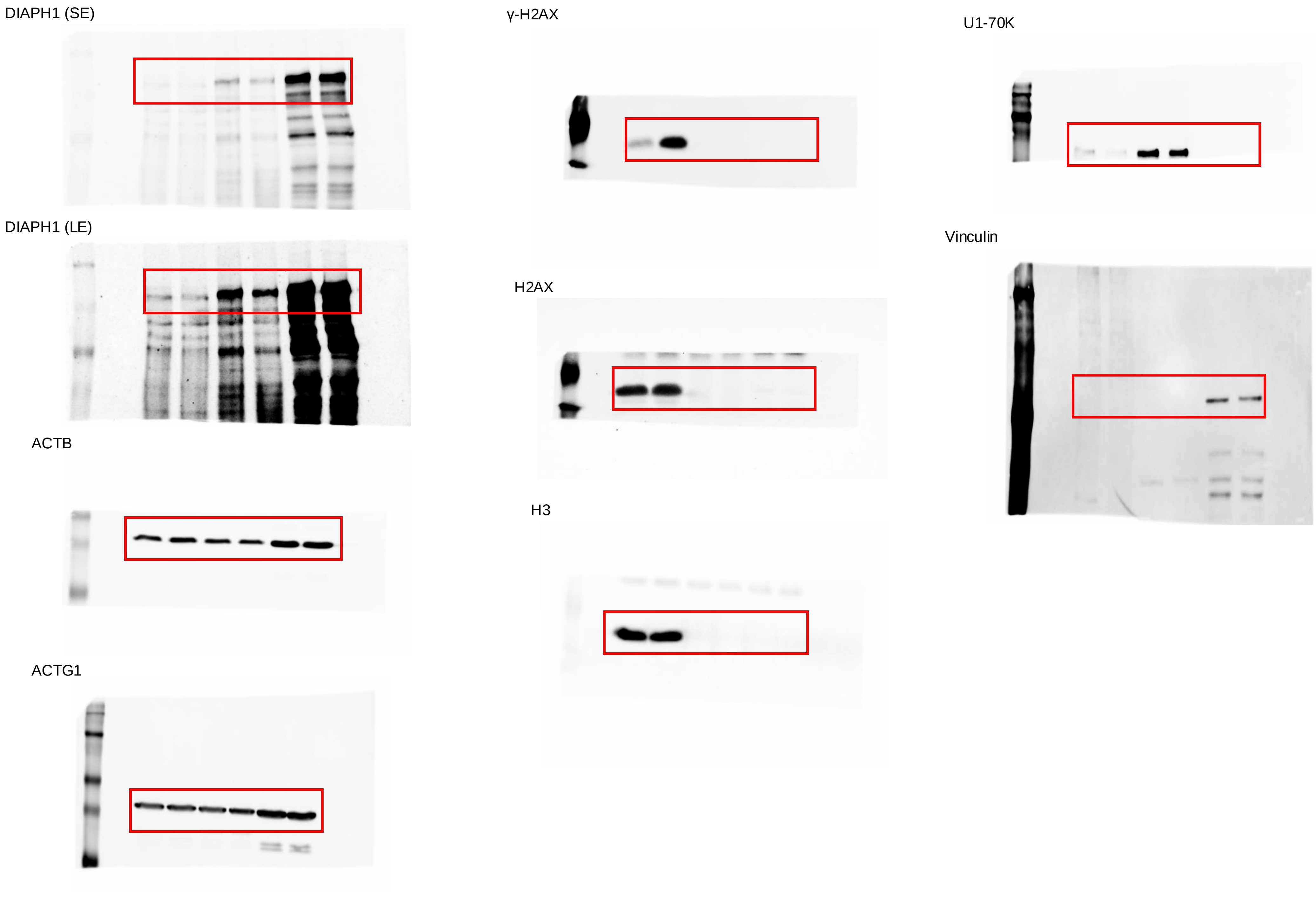

Figure S8b

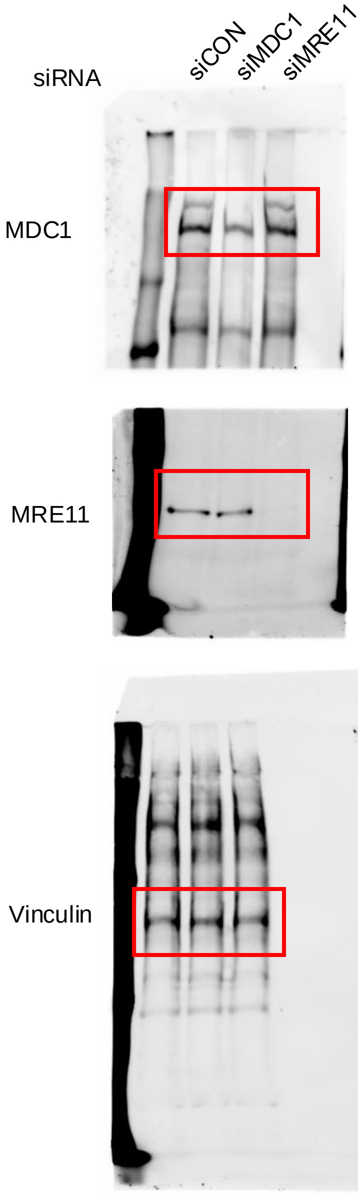

Figure S13A

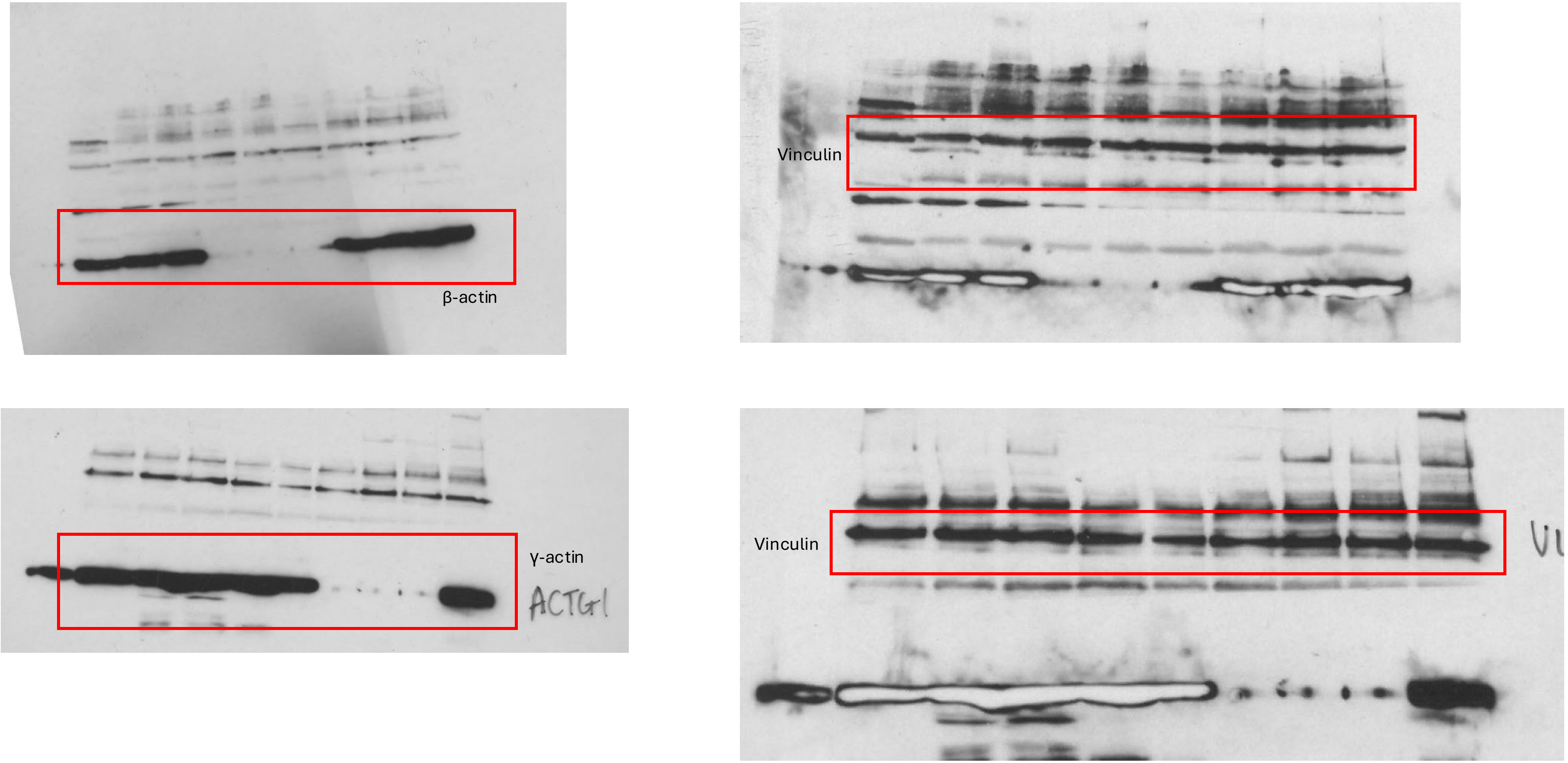

Figure S16C

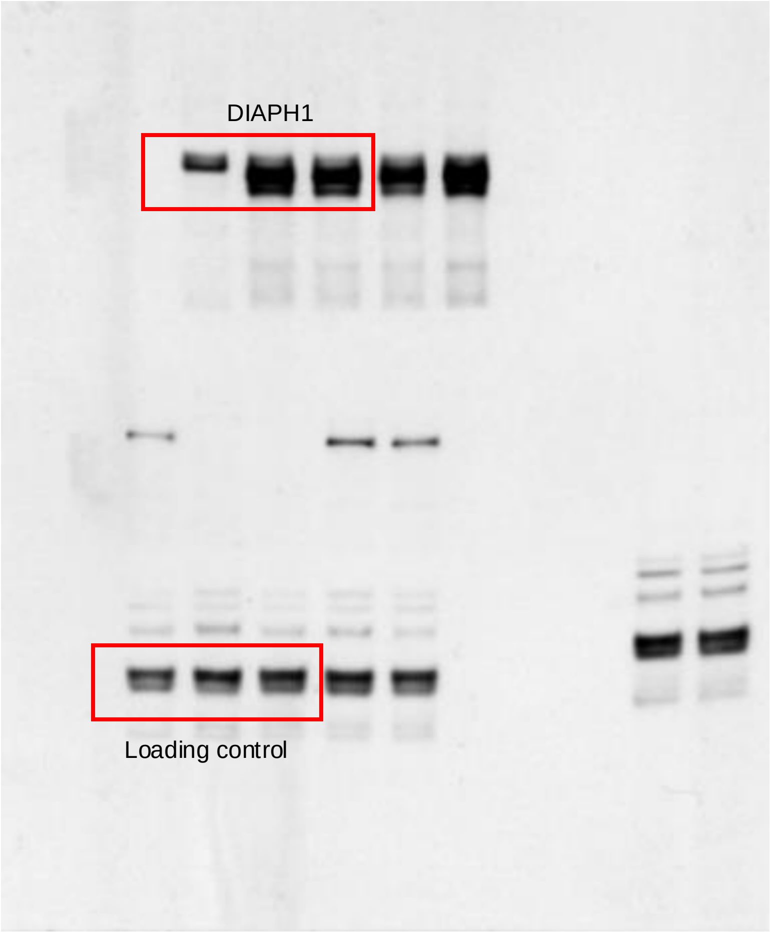

Figure S17A

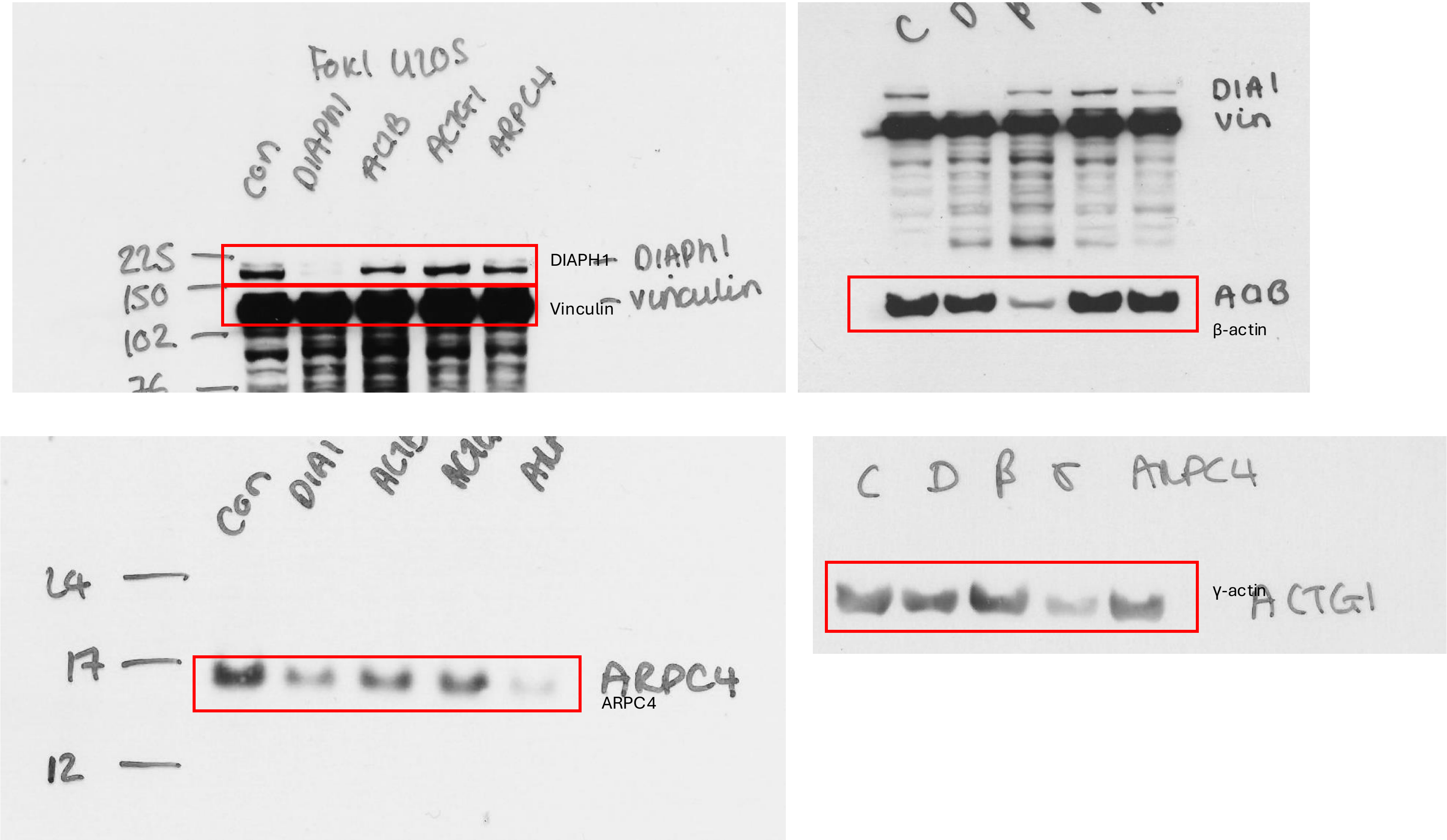

Figure S17B

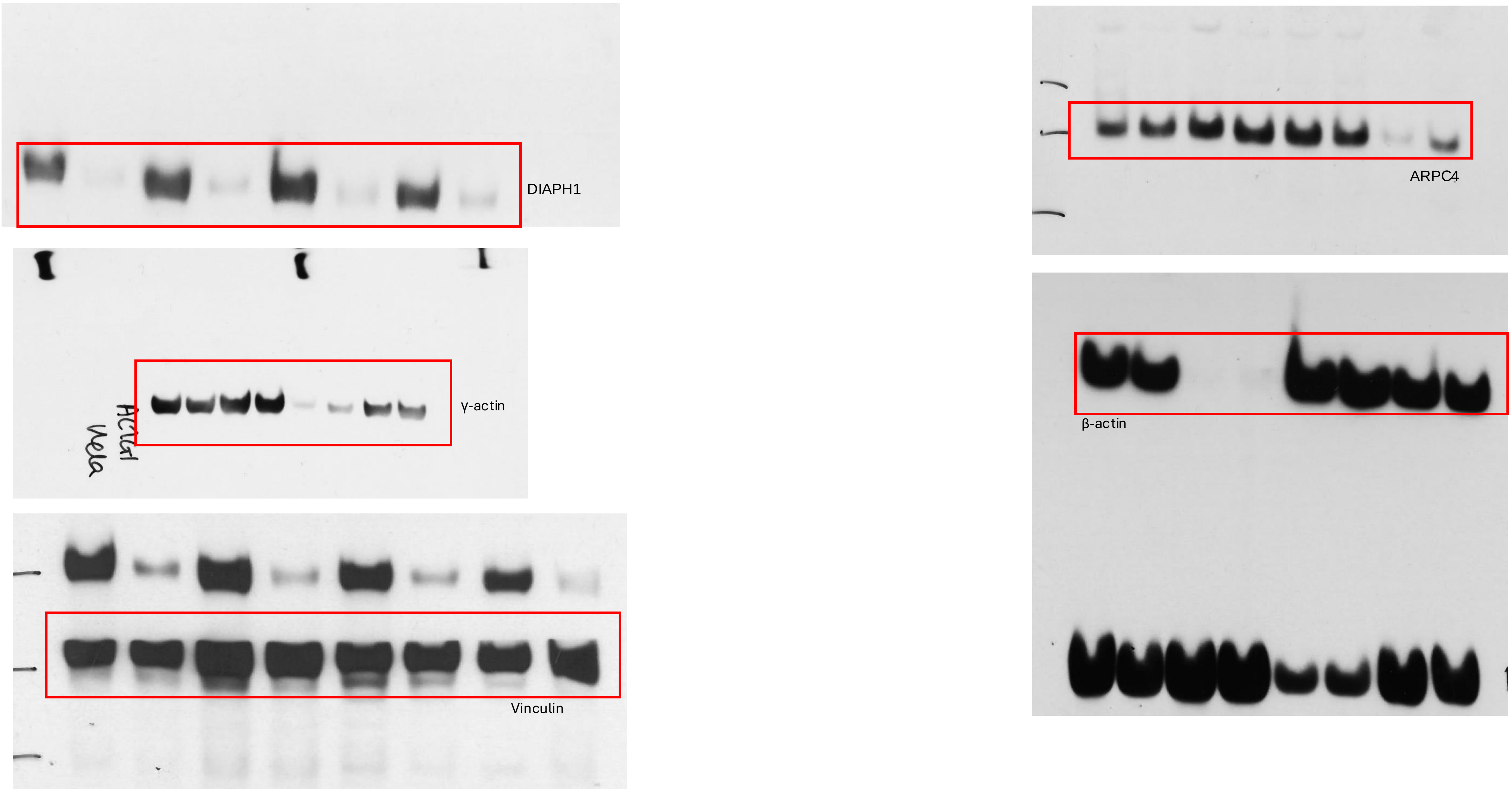

Supplement: Supplementary file 4 — Source Data [file 41467_2025_59553_MOESM4_ESM.zip › Source_data_Western_blots.pdf]
